# Structural Evolution and Electronic Properties of Selenium-Doped Boron Clusters SeB_n_^0/−^ (n = 3–16)

**DOI:** 10.3390/molecules28010357

**Published:** 2023-01-01

**Authors:** Yue-Ju Yang, Shi-Xiong Li, De-Liang Chen, Zheng-Wen Long

**Affiliations:** 1 School of Physics and Electronic Science, Guizhou Education University, Guiyang 550018, China; 2 College of Physics, Guizhou University, Guiyang 550025, China

**Keywords:** boron clusters, geometrical structures, photoelectron spectra

## Abstract

A theoretical research of structural evolution, electronic properties, and photoelectron spectra of selenium-doped boron clusters SeB_n_^0/−^ (n = 3–16) is performed using particle swarm optimization (CALYPSO) software in combination with density functional theory calculations. The lowest energy structures of SeB_n_^0/−^ (n = 3–16) clusters tend to form quasi-planar or planar structures. Some selenium-doped boron clusters keep a skeleton of the corresponding pure boron clusters; however, the addition of a Se atom modified and improved some of the pure boron cluster structures. In particular, the Se atoms of SeB_7_^−^, SeB_8_^−^, SeB_10_^−^, and SeB_12_^−^ are connected to the pure quasi-planar B_7_^−^, B_8_^−^, B_10_^−^, and B_12_^−^ clusters, which leads to planar SeB_7_^−^, SeB_8_^−^, SeB_10_^−^, and SeB_12_^−^, respectively. Interestingly, the lowest energy structure of SeB_9_^−^ is a three-dimensional mushroom-shaped structure, and the SeB_9_^−^ cluster displays the largest HOMO–LUMO gap of 5.08 eV, which shows the superior chemical stability. Adaptive natural density partitioning (AdNDP) bonding analysis reveals that SeB_8_ is doubly aromatic, with 6 delocalized π electrons and 6 delocalized σ electrons, whereas SeB_9_^−^ is doubly antiaromatic, with 4 delocalized π electrons and 12 delocalized σ electrons. Similarly, quasi-planar SeB_12_ is doubly aromatic, with 6 delocalized π electrons and 14 delocalized σ electrons. The electron localization function (ELF) analysis shows that SeB_n_^0/−^ (n = 3–16) clusters have different local electron delocalization and whole electron delocalization effects. The simulated photoelectron spectra of SeB_n_^−^ (n = 3–16) have different characteristic bands that can identify and confirm SeB_n_^−^ (n = 3–16) combined with future experimental photoelectron spectra. Our research enriches the geometrical structures of small doped boron clusters and can offer insight for boron-based nanomaterials.

## 1. Introduction

Clusters are composed of several to thousands of atoms or molecules whose properties depend on their size and shape. Clusters are ideal model systems for correlating microscopic structure and macroscopic properties of substances, and cluster research is of great significance to deeply understand the laws of matter transformation. Research of structure and properties of clusters can offer insight for the design and manufacture of new materials and new devices at the atomic level. Boron clusters can induce polycentric chemical bonds and adopt several interesting structures with meaningful properties [1,2,3,4,5,6]. Experimental and theoretical research has shown that B_n_^−^ (n < 38) have quasi-planar or planar structures [7,8], and neutral B_n_ have quasi-planar, planar, wheel shaped, tubular structures or other structures [1,5,9,10,11]. In 2014, the experimental finding of cage-type all-boron cluster (borospherene) [12] B_40_^−^ has given rise to a lot of attention on boron clusters [13,14,15,16,17,18,19,20,21]. In 2015, researchers synthesized borophene on the Ag (111) base [22], and the structural unit of borophene is a B_7_ cluster. In 2021, researchers synthesized borophene crystal, which was hydrogenated with hydrogen atoms [23], and it is very stable and comparable to graphene. Interestingly, the basic unit of hydrogenated borophene happens to be a hydrogenated B_7_ cluster also. The experimental findings of B_40_^−^ and borophene offer insight for the development of new boron nanomaterials and nanodevices. Research on small boron clusters is promising as a way to provide new ideas for new nanomaterials and nanodevices.

In recent years, researchers have studied abundant doped boron clusters, and they mainly focused on doping a single metal atom in boron clusters of different sizes. Metal-atom-doped boron clusters can induce new geometrical configuration and properties [15,17,18,19,24,25,26,27,28,29,30,31,32,33,34,35,36,37,38]. For example, anionic B_20_^−^ and B_22_^−^ have a quasi-planar structure [7,39]. However, single alkali-metal-atom-doped LiB_20_^−^, NaB_22_^−^, and KB_22_^−^ display a double-ring configuration [25,40]. Co and Rh atom-doped boron clusters MB_12_ (M = Co and Rh) can lead to the quasi-planar B_12_ form, a semi-sandwich configuration [3,24]. Neutral B_24_ has a double-ring configuration [39], while TiB_24_ and ScB_24_ have a cage configuration and three-ring tubular structure, respectively, after adding one Sc or Ti atom [41,42]. Anionic B_24_^−^ has a quasi-planar structure [7], while TiB_24_^−^ and VB_24_^−^ have a cage configuration after adding one Ti or V atom [43]. In addition, the LiB_40_, NaB_40_, or KB_40_ cage is promising for application to the field of nonlinear optics [18]; the ScB_40_ or TiB_40_ cage is promising for application to the field of hydrogen storage [15,17,19]; Co atom-doped CoB_12_^−^ and Rh atom-doped RhB_12_^−^ can improve chemical activity [27]; Co atom-doped CoB_40_ is promising for application to molecular devices [26]; and the metal-atom-doped boron clusters ReB_n_^−^(n = 3–4, 6, 8–9), MnB_n_^−^(n = 6, 16), BiB_n_^−^(n = 6–8), CoB_16_^−^, La_2_B_n_^−^(n = 10–11), La_3_B_18_^−^, MB_8_(M = Be, Mg), and M_2_B_6_(M = Mg, Ca, Sr) have various unique structures [29,30,31,32,33,34,35,36,37,44,45]. However, nonmetallic-atom-doped boron clusters have been poorly studied. In particular, the structural evolution of boron clusters after addition of a single nonmetallic atom is rarely studied. Similar to B_7_, small boron clusters with doping are promising as the structural units of borophene and other boron nanomaterials. Selenium is one of the essential microelements in the human body and has obvious inhibitory effects on tumors. The Se atom can combine with metal atoms, such as Cd and Zn, to form semiconductor clusters or quantum dots [46,47]. These materials exhibit a variety of unique optical and electronic properties, which can be further applied in imaging and diagnosis of biological systems [48,49]. Se doping of boron clusters is promising to be a useful strategy to further increase the diversity of structural forms and to affect the properties of boron clusters. Therefore, the theoretical research of Se atom-doped small boron clusters can enrich new structures and new properties of boron clusters, and can also provide theoretical guidance for the synthesis of nanomaterials, such as borophene. Herein, to demonstrate the structural evolution of Se-doped SeB_n_^0/−^ (n = 3–16), extensive geometric configurations were generated and predicted, using the particle swarm optimization (CALYPSO) approach [50] in combination with the density functional theory method PBE0 [51].

## 2. Results and Discussion

### 2.1. Structures and Electronic Properties

**Figure 1:**
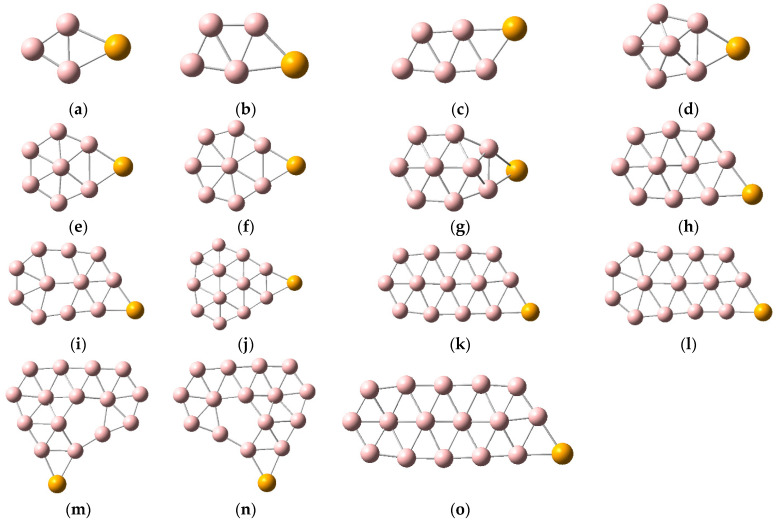
Structures of SeB_n_. (**a**) SeB_3_ C_2V_; (**b**) SeB_4_ Cs; (**c**) SeB_5_ Cs; (**d**) SeB_6_ Cs; (**e**) SeB_7_ Cs; (**f**) SeB_8_ C_2V_; (**g**) SeB_9_ Cs; (**h**) SeB_10_; (**i**) SeB_11_ Cs; (**j**) SeB_12_ Cs; (**k**) SeB_13_ C_1_; (**l**) SeB_14_ Cs; (**m**) SeB_15_ I C_1_; (**n**) SeB_15_ II C_1_; (**o**) SeB_16_ C_1_.

**Figure 2:**
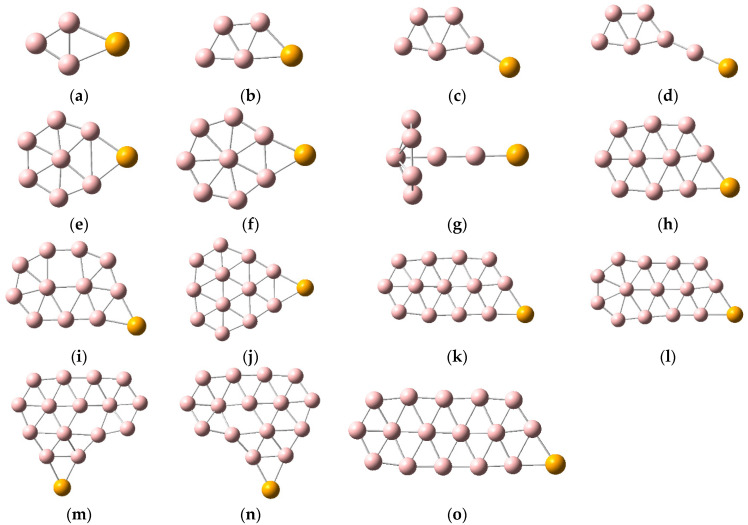
Structures of SeB_n_^−^. (**a**) SeB_3_^−^ C_2V_; (**b**) SeB_4_^−^ Cs; (**c**) SeB_5_^−^ Cs; (**d**) SeB_6_^−^ C_1_; (**e**) SeB_7_^−^ C_2V_; (**f**) SeB_8_^−^ C_2V_; (**g**) SeB_9_^−^ C_6V_; (**h**) SeB_10_^−^ C_1_; (**i**) SeB_11_^−^ Cs; (**j**) SeB_12_^−^ C_2V_; (**k**) SeB_13_^−^ C_1_; (**l**) SeB_14_^−^ Cs; (**m**) SeB_15_^−^ I C_1_; (**n**) SeB_15_^−^ II C_1_; (**o**) SeB_16_^−^ C_1_.

Five low-energy structures of SeB_n_^0/−^ (n = 3–16) are shown in Figures S1–S28, and the lowest energy structures of SeB_n_^0/−^ (n = 3–16) are displayed in Figure 1 and Figure 2. The results indicate that the low-energy structures of SeB_n_^0/−^ (n = 3–16) trend to form planar or quasi-planar structures. Interestingly, the ground state configuration of SeB_9_^−^ is three-dimensional mushroom shaped. Early theoretical and experimental research found that most of the small neutral boron clusters are quasi-planar or planar, and all small anionic boron clusters are quasi-planar or planar structures. Figure 1 and Figure 2 and the research results indicate that, after adding a Se atom, some of the lowest energy configurations of SeB_n_^0/−^ (n = 3–16) have a skeleton of pure boron clusters, such as SeB_n_ (n = 3–5, 7–8, 10–14, 16) and SeB_n_^−^ (n = 3–5, 7–8, 10–14, 16) [52,53]. As can be seen in Figure 1 and Figure 2, except for SeB_n_^−^ (n = 5, 6, 9), each Se atom is connected to two boron atoms to form a three-ring. The lowest energy configurations of SeB_n_^0/−^ (n = 3–5) have planar structure, and the Se atom is attached to the boron atoms of the pure boron cluster B_n_^0/−^ (n = 3–5) [52]. The lowest energy configurations of SeB_6_^0/−^ and SeB_13_^0/−^ have quasi-planar structure, and they are different from the ground-state structures of corresponding pure boron clusters B_6_^0/−^ and B_13_^0/−^ [52]. For SeB_7_^0/−^, SeB_10_^0/−^, and SeB_12_^0/−^, the lowest energy configurations of neutral clusters and corresponding anionic clusters have similar structure, and the Se atoms of SeB_7_, SeB_10_, and SeB_12_ are connected to the pure quasi-planar B_7_, B_10_, and B_12_ clusters, respectively. However, the Se atoms of SeB_7_^−^, SeB_10_^−^, and SeB_12_^−^ are connected to the pure quasi-planar B_7_^−^, B_10_^−^, and B_12_^−^ clusters, which leads to the planar SeB_7_^−^, SeB_10_^−^, and SeB_12_^−^, respectively [52]. The lowest energy structures of SeB_8_^0/−^ have same planar structure, and the Se atom of SeB_8_ is connected to the pure planar B_8_ cluster. However, the Se atom of SeB_8_^−^ is connected to the pure quasi-planar B_8_^−^ cluster, which leads to the planar SeB_8_^−^. The pure B_9_^0/−^ have same planar wheel-shape structure [52]. However, doping of Se atom causes the anionic SeB_9_^−^ to become a three-dimensional mushroom-shaped structure (with C_6V_ symmetry) and causes the neutral SeB_9_ to become a boat-shaped structure. The lowest energy structures of SeB_11_^0/−^ have similar structure, and the Se atom of SeB_11_ is connected to the pure planar B_11_ cluster, which leads to the slight structural change. However, the Se atom of SeB_11_^−^ is connected to the pure planar B_11_^−^ cluster [52]. The lowest energy configurations of SeB_14_^0/−^ have same planar structure, the lowest energy configurations of SeB_16_^0/−^ have same quasi-planar structure, and the Se atoms are connected to the pure planar B_14_^0/−^ clusters and pure quasi-planar B_16_^0/−^ clusters, respectively [52,53]. The lowest energy configurations of SeB_15_^0/−^ have quasi-planar structure and exhibit two axially chiral isomers, and they are different from the ground-state configurations of pure boron cluster B_15_^0/−^. Similar to pure B_7_ clusters, planar and quasi-planar Se-doped boron clusters are promising as the structural units of boron nanomaterials, which can be synthesized further into borophene.

The harmonic frequency analysis confirmed that these lowest-energy structures are actually stable (no imaginary frequency). For closed-shell clusters, the highest occupied molecular orbital (HOMO) and the lowest unoccupied molecular orbital (LUMO) energy gaps (HOMO–LUMO energy gaps) of SeB_3_^−^, SeB_4_, SeB_5_^−^, SeB_6_, SeB_7_^−^, SeB_8_, SeB_9_^−^, SeB_10_, SeB_11_^−^, SeB_12_, SeB_13_^−^, SeB_14_, SeB_15_^−^, and SeB_16_ are 2.48, 3.52, 2.14, 3.92, 2.90, 2.33, 5.08, 2.93, 2.89, 3.29, 2.28, 2.00, 2.91, and 2.27 eV, respectively. For open-shell clusters, α-HOMO–LUMO and β-HOMO–LUMO, energy gaps vary within the range of 1.92-4.39 eV. Meanwhile, the calculated HOMO–LUMO energy gaps of SeB_n_^0/−^ (n = 3–16) clusters reveal that the SeB_9_^−^ cluster possesses the largest HOMO-LUMO gap of 5.08 eV, which shows the superior chemical stability.

**Figure 3:**
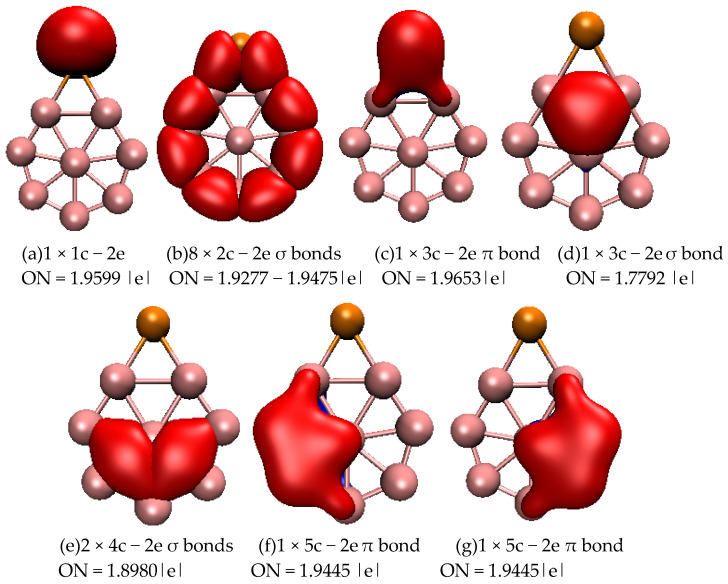
Bonding patterns of SeB_8_. ON is occupation number and the orange ball is Se atom. (**a**) 1 × 1c − 2e, ON = 1.9599 |e|; (**b**) 8 × 2c − 2e σ bonds, ON = 1.9277 − 1.9475 |e|; (**c**) 1 × 3c − 2e π bond, ON = 1.9653 |e|; (**d**) 1 × 3c − 2e σ bond, ON = 1.7792 |e|; (**e**) 2 × 4c − 2e σ bonds, ON = 1.8980 |e|; (**f**) 1 × 5c − 2e π bond_,_ ON = 1.9445 |e|; (**g**) 1 × 5c − 2e π bond, ON = 1.9445 |e|.

**Figure 4:**
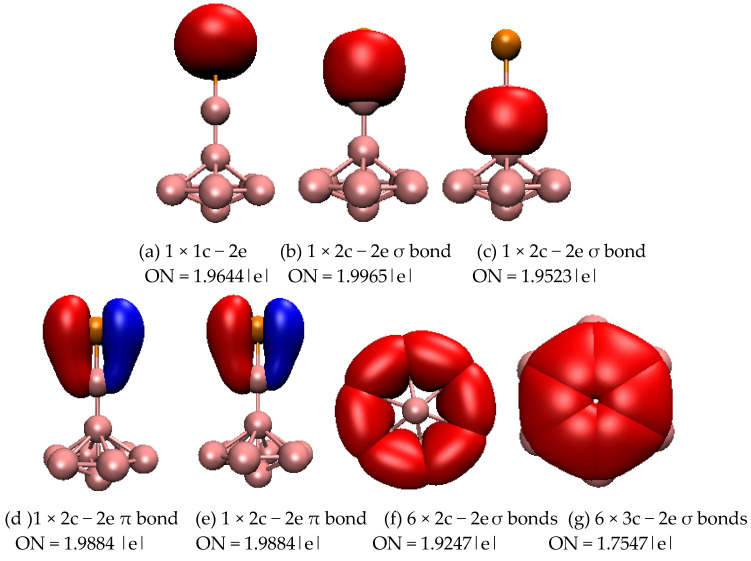
Bonding patterns of SeB_9_^−^. ON is occupation number and the orange ball is Se atom. (**a**) 1 × 1c − 2e, ON = 1.9644 |e|; (**b**) 1 × 2c − 2e σ bond, ON = 1.9965 |e|; (**c**) 1 × 2c − 2e σ bond, ON = 1.9523 |e|; (**d**) 1 × 2c − 2e π bond, ON = 1.9884 |e|; (**e**) 1 × 2c − 2e π bond, ON = 1.9884 |e|; (**f**) 6 × 2c − 2e σ bonds_,_ ON = 1.9247 |e|; (**g**) 6 × 3c − 2e σ bonds, ON = 1.7547 |e|.

**Figure 5:**
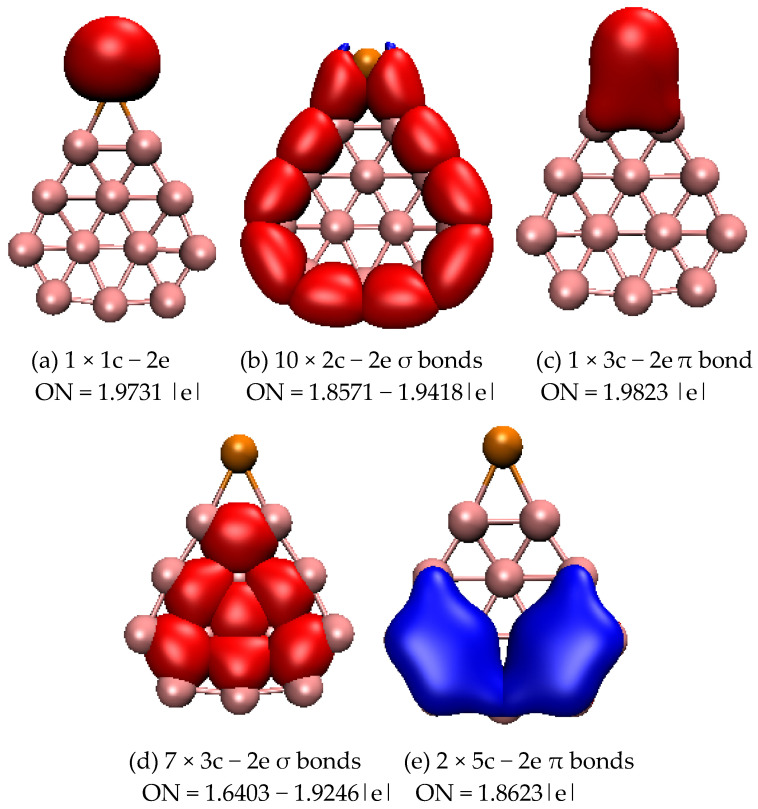
Bonding patterns of SeB_12_. ON is occupation number and the orange ball is Se atom. (**a**) 1 × 1c − 2e, ON = 1.9731 |e|; (**b**) 10 × 2c − 2e σ bonds, ON = 1.8571 − 1.9418 |e|; (**c**) 1 × 3c − 2e π bond, ON = 1.9823 |e|; (**d**) 7 × 3c − 2e σ bonds, ON = 1.6403 − 1.9246 |e|; (**e**) 2 × 5c − 2e π bonds, ON = 1.8623 |e|.

To further understand the stability of typical structures of SeB_n_^0/−^ (n = 3–16), we analyzed the chemical bonding of closed-shell planar SeB_8_, three-dimensional SeB_9_^−^, and quasi-planar SeB_12_ using the adaptive natural density partitioning (AdNDP) approach. Figure 3 displays the bonding patterns of planar SeB_8_. For SeB_8_, AdNDP analyses reveal that one lone pair (Figure 3a) is found on the Se atom and eight 2c–2e σ bonds (Figure 3b) on the peripheral ring. The remaining six bonds contain three σ bonds and three π bond, which are classified into four sets (Figure 3): one 3c–2e σ bond covers one B_3_ triangle, in which two of the boron atoms are connected to the Se atoms; two 4c–2e σ bonds cover two B_4_ rings; one 3c–2e π bond is distributed around the B-Se-B triangle; and two 5c–2e π bonds cover two B_5_ rings. Overall, the eight 2c–2e σ bonds, one delocalized 3c–2e σ bond, and two delocalized 4c–2e σ bonds cover the planar molecule, which renders stability to the SeB_8_, and the delocalized 3c–2e π bond and 5c–2e π bonds further stabilize the SeB_8_ cluster. Figure 4 displays the bonding patterns of SeB_9_^−^, and there are five categories. First, there is one lone pair (see Figure 4a) on the Se atom. Then, there are two 2c–2e σ bonds on the Se-B and adjacent B-B. Third, two 2c–2e π bonds cover the Se-B symmetrically. Fourth, the peripheral B_7_ ring at the top of the mushroom is characterized by six localized B−B 2c–2e σ bonds. The last six delocalized 3c–2e σ bonds cover the inner B_3_ triangles at the top of the mushroom. The six localized B−B 2c-2e σ bonds and six delocalized 3c–2e σ bonds are responsible for the connection between the outer B_7_ ring and the inner B atom at the top of the mushroom, which enhances the stability of SeB_9_^−^. Figure 5 displays the bonding patterns of quasi-planar SeB_12_. For SeB_12_, AdNDP analyses reveal that one lone pair (see Figure 5a) is found on the Se atom and ten 2c–2e σ bonds (see Figure 5b) on the peripheral ring. The remaining ten bonds contain seven σ bonds and three π bonds, which are classified into three sets (Figure 5): one 3c–2e π bond covers B-Se-B triangle, seven 3c–2e σ bonds cover the seven inner B_3_ triangles, and two 5c–2e π bonds are distributed symmetrically around the two B_5_ rings. Similar to the SeB_8_, the ten 2c–2e σ bonds and seven delocalized 3c–2e σ bonds cover the quasi-planar molecule, which renders stability to the SeB_12_ cluster, and the delocalized 3c–2e π bond and 5c–2e π bonds further stabilize the SeB_12_ cluster. AdNDP bonding analyses revealed that the SeB_8_ and SeB_12_ possess three delocalized π bonds, which, quite surprisingly, satisfy the 4m + 2 Hückel rule for π aromaticity. Furthermore, SeB_8_ and SeB_12_ possess three delocalized σ bonds and seven delocalized σ bonds, which satisfy the 4m + 2 Hückel rule for σ aromaticity. However, the three-dimensional SeB_9_^−^ cluster possesses two delocalized π bonds and six delocalized σ bonds, which satisfy the 4m Hückel rule for π and σ antiaromaticity.

To describe the electron localization or delocalization of electrons, the electron localization function (ELF) [54] of the valence electrons was analyzed, as shown in Figures S29–S31. At the isosurface value of 0.60, the isosurface maps of most of the clusters are connected on the surface of the whole molecule. Yet, the isosurface diagrams of SeB_5_^−^, SeB_6_^−^, SeB_9_^−^, and SeB_11_^−^ are disconnected on the surface of the whole molecule, indicating that the delocalization of the whole molecule is weaker than that of the other clusters. Figure S30 displays the ELF with the isosurface value of 0.70. The isosurface diagram of SeB_3_, SeB_3_^−^, SeB_4_^−^, SeB_9_, SeB_12_, and SeB_15_^−^ is still connected on the surface of the whole molecule, while the isosurface diagrams of other clusters are broken on the partial regions of the molecule, indicating that the delocalization of SeB_3_, SeB_3_^−^, SeB_4_^−^, SeB_9_, SeB_12_, and SeB_15_^−^ is stronger than that of the other clusters. Figure S31 displays the ELF with the isosurface value of 0.80, in which the isosurface maps of some clusters are disconnected and there are no connected regions. The isosurface maps of SeB_3_, SeB_3_^−^, SeB_6_, SeB_7_, SeB_7_^−^, SeB_9_^−^, SeB_12_, and SeB_15_^−^ show that there is still some connected area on the surface of molecule, indicating that the local delocalization of these clusters is stronger than that of other clusters. Quite specially, the isosurface diagram of SeB_9_^−^ is still connected on the peripheral B_7_ ring at the top of the mushroom. ELF analyses further confirm these observations based on the AdNDP analyses, such as the contributions from the valence electrons of the SeB_12_ were partitioned in Figure S31. Isosurface maps of the SeB_12_ (Figure S31) cover eight peripheral B-B bonds and two B-Se bonds that correspond to ten peripheral 2c–2e σ bonds, and they cover seven B_3_ triangles that correspond to seven 3c–2e σ bonds. Isosurface maps on the two B_5_ ring are fatter due to another two 5c–2e π bonds.

Figure S32 shows the isosurface diagram of the spin density of the open-shell clusters, and spin density can reveal the distribution of unpaired electrons. Figure S32 shows the spin density diagram with an isosurface value of 0.002, in which green represents alpha electrons and blue represents beta electrons. Figure S32 shows that the unpaired single electrons are mostly alpha electrons, and there are a small number of beta electrons on B atoms. Most of the unpaired alpha electrons are distributed on the B atoms; only a small portion of the unpaired alpha electrons are distributed on the Se atom. The spin density can reflect chemical reactions or adsorption to a certain extent. The single electrons of these clusters are mostly alpha electrons and are basically on the B atoms. The B or Se atoms with single alpha electrons can pair with free radicals or small molecules with beta single electrons to form new covalent bonds. In addition, these spin features are expected to produce interesting magnetic properties, which will further lead to potential applications in molecular devices.

### 2.2. Photoelectron Spectra

Photoelectron spectroscopy in combination with theoretical calculations was used to identify the structures of size-selected boron clusters [3,12,55]. To assist with future identifications of SeB_n_^−^ (n = 3–16), vertical detachment energies (VDEs) were calculated and photoelectron spectra of SeB_n_^−^ (n = 3–16) were simulated with the time-dependent DFT (TD-DFT) method [12,55,56].

**Figure 6:**
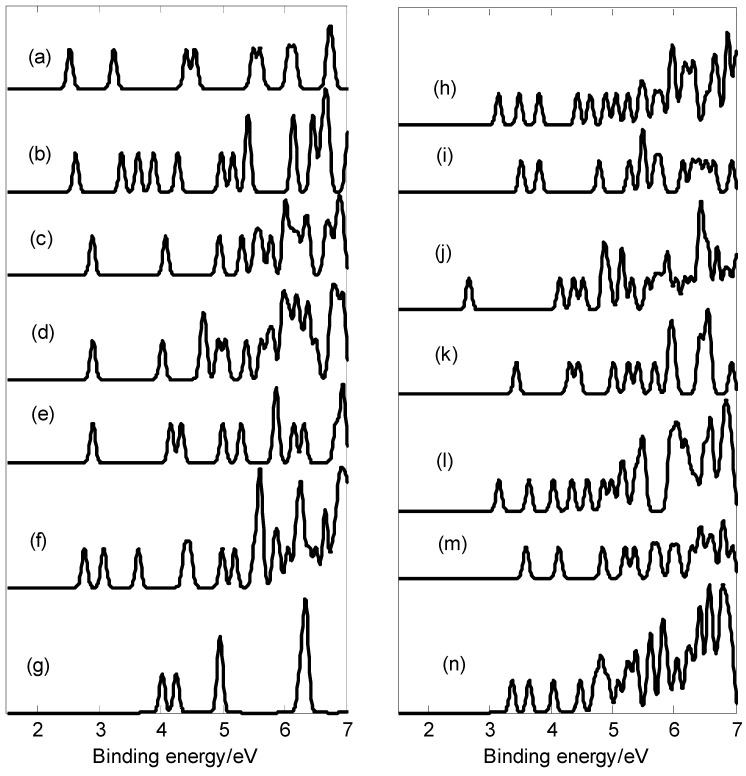
Photoelectron spectra at the PBE0/6-311 + G * level. (**a**) SeB_3_^−^; (**b**) SeB_4_^−^; (**c**) SeB_5_^−^; (**d**) SeB_6_^−^; (**e**) SeB_7_^−^; (**f**) SeB_8_^−^; (**g**) SeB_9_^−^; (**h**) SeB_10_^−^; (**i**) SeB_11_^−^; (**j**) SeB_12_^−^; (**k**) SeB_13_^−^; (**l**) SeB_14_^−^; (**m**) SeB_15_^−^; (**n**) SeB_16_^−^.

Figure 6 presents the photoelectron spectra of SeB_n_^−^ (n = 3–16). The results indicate that SeB_3_^−^ has the lowest first VDE, and SeB_12_^−^ has the largest energy gap (about 1.48 eV) between the first and second peaks. The first several peaks were used to identify boron clusters [3,12]; the peaks on the low binding energy side are of great significance. The first peaks of these photoelectron spectra (except for SeB_9_^−^) come from the calculated ground-state VDEs of SeB_3_^−^, SeB_4_^−^, SeB_5_^−^, SeB_6_^−^, SeB_7_^−^, SeB_8_^−^, SeB_10_^−^, SeB_11_^−^, SeB_12_^−^, SeB_13_^−^, SeB_14_^−^, SeB_15_^−^, and SeB_16_^−^ at 2.52, 2.62, 2.89, 2.89, 2.89, 2.76, 3.14, 3.51, 2.66, 3.43, 3.15, 3.59, and 3.36 eV, respectively. The calculated ground-state VDEs of these closed-shell clusters originate from the detachment of the electron from the molecular orbital HOMO. However, for open-shell clusters, the calculated ground-state VDE of each cluster originates from the detachment of the electron from the singly occupied molecular orbital *α*-SOMO. The first peak of SeB_9_^−^ comes from the second VDE at 4.01 eV, which is smaller than the ground-state VDE of 4.25 eV (second peak). The second peaks of SeB_3_^−^, SeB_5_^−^, SeB_7_^−^, SeB_11_^−^, SeB_13_^−^, and SeB_15_^−^ come from the second calculated VDEs at 3.23, 4.07, 4.16, 3.81, 4.30, and 4.12 eV, respectively, which originate from detaching the electrons from HOMO-1. The second peaks of SeB_4_^−^, SeB_6_^−^, SeB_8_^−^, SeB_10_^−^, SeB_12_^−^, SeB_14_^−^, and SeB_16_^−^ come from the second VDEs at 3.36, 4.02, 3.07, 3.48, 4.14, 3.64, and 3.63 eV, respectively, which originate from detaching the electrons from the singly occupied molecular orbital β -HOMO-1. In addition, the peaks with higher binding energy originate from detaching the electrons from lower molecular orbitals. It is noted that some of the doped anionic boron clusters have a similar skeleton as the corresponding anionic pure boron clusters. Comparing their photoelectron spectra, the addition of the Se atom results in a great change in the photoelectron spectra [52,53]. However, the photoelectron spectra of some doped boron clusters are similar to those of the corresponding anionic pure boron clusters [52,53]. For example, compared with the photoelectron spectra of pure boron clusters, the addition of Se atoms causes the first two peaks of SeB_3_^-^ to move 0.30 eV towards the low binding energy side and causes the first two peaks of B_5_^-^ to move 0.46 eV towards the high binding energy side [52]. For SeB_4_^−^, SeB_8_^−^, and SeB_13_^-^, compared with the photoelectron spectra of pure boron clusters, the addition of Se atoms causes different band characteristics [52]. Compared to the photoelectron spectra of pure boron clusters, planar SeB_7_^−^ and quasi-planar B_7_^-^ have almost the same first VDE (2.89 eV for SeB_7_^-^, 2.85 ± 0.02 eV for B_7_^−^) [52], planar SeB_10_^-^ and quasi-planar B_10_^-^ have almost the same first VDE (3.14 eV for SeB_10_^−^, 3.06 ± 0.03 eV for B_10_^−^) [52], and quasi-planar SeB_14_^−^ and quasi-planar B_14_^-^ have almost the same first VDE (3.14 eV for SeB_14_^−^, 3.10 ± 0.01 eV for B_14_^−^) [52]. For SeB_11_^-^, compared to the photoelectron spectra of pure boron clusters B_11_^−^, the addition of the Se atom causes the first peak to move 0.08 eV towards the high binding energy side and causes the second peak to move 0.25 eV towards the low binding energy side. For SeB_12_^−^ and SeB_16_^−^, planar SeB_12_^-^ and quasi-planar B_12_^−^ have similar band characteristics [52], and quasi-planar SeB_16_^−^ and quasi-planar B_16_^-^ have similar band characteristics [53]. Figure 6 indicates that SeB_n_^−^ (n = 3–16) has different spectral features; especially the peaks at the low binding energy side can identify the SeB_n_^−^ (n = 3–16). As with the discovery of other anionic boron clusters, if the photoelectron spectra of SeB_n_^−^ (n = 3–16) are obtained in experiments, these simulated values can be used for the identification of SeB_n_^−^ (n = 3–16).

## 3. Computation Details

Configuration searches of Se-doped boron clusters SeB_n_^0/−^ (n = 3–16) were performed with CALYPSO 5.0 software in combination with Gaussian 16 software. CALYPSO is a reliable cluster configuration prediction software, and it has successfully predicted boron or doped boron clusters [11,25,40,41,45,57,58,59,60,61]. The initial structures were generated by the CALYPSO software, and then these initial structures were optimized using Gaussian 16 software at the PBE0/3-21G level for the preliminary structural search. In each generation produced by the CALYPSO software, 70% of the structures were produced by particle swarm optimization (PSO) operations, while the others were randomly generated. When cluster sizes vary from n = 3 to n = 10, nearly 100–900 isomers are initially predicted for each boron cluster of a different size. When cluster sizes vary from n = 11 to n = 16, nearly 2000 isomers are initially predicted for each boron cluster of a different size.

After the preliminary structural search, low-energy structures were then fully optimized at the PBE0/6-311+G(d) level [51,62]. After the optimizations, frequency analyses and electronic structures were studied at the PBE0/6-311+G(d) level. PBE0/6-311+G(d) is a reliable level for boron cluster [12,60,61,63,64,65]; in particular, theoretical simulated values with PBE0/6-311+G(d) are the same as the experimental values [12]. Therefore, all calculations in this article used the method PBE0/6-311+G(d) and were performed using Gaussian 16 software [66]. The analyses and isosurface map drawings were performed using Multiwfn 3.7 code [67] and the visual molecular dynamics (VMD) program [68].

## 4. Conclusions

DFT combined with CALYPSO software is employed to demonstrate the structural evolution of SeB_n_^0/−^ (n = 3–16) clusters. The conclusions are summarized as follows. (1) The global minima of SeB_n_^0/−^ (n = 3–16) clusters tend to form quasi-planar or planar structures. (2) The ground-state structure of SeB_9_^−^ is a three-dimensional, mushroom-shaped, ground-state structure, and it possesses the largest HOMO–LUMO gap of 5.08 eV, which shows the superior chemical stability. (3) AdNDP bonding analyses reveal that SeB_8_ is doubly aromatic, with six delocalized σ and six delocalized π electrons, whereas SeB_9_^−^ is doubly antiaromatic, with twelve delocalized σ and four delocalized π electrons. Similarly, SeB_12_ is doubly aromatic, with fourteen delocalized σ and six delocalized π electrons. (4) ELF analysis shows that SeB_n_^0/−^ (n = 3–16) clusters have different local electron delocalization and whole-electron delocalization effects. (5) SeB_n_^−^ (n = 3–16) have different photoelectron spectra, and especially the first several peaks can be used for the identification of SeB_n_^−^ (n = 3–16). This research has enriched the structures of doped boron clusters.

## Data Availability

The data presented in this study are available in the article and Supplementary Materials.

## Supplementary Materials

The following supporting information can be downloaded at: https://www.mdpi.com/article/10.3390/molecules28010357/s1, Figures S1–28: Low-lying isomers of doped boron clusters SeB_n_^0/−^ (n = 3–16); Figures S29–31: Electron localization function (ELF) of SeB_n_^0/−^ (n = 3–16); Figure S32: Spin density of open-shell doped boron clusters.

## Sample Availability

Samples of the compounds are available from the authors.
