# Supplementary material for: Structural Evolution and Electronic Properties of Selenium-Doped Boron Clusters SeBn0/− (n = 3–16)
Source: Molecules. 2023 Jan 1;28(1):357. doi: 10.3390/molecules28010357 (PMC9824103; doi:10.3390/molecules28010357)
Supplement: Supplementary file 1 [file molecules-28-00357-s001.zip › molecules-2055645-supplementary.pdf]

Structural Evolution and Electronic Properties of Selenium-Doped Boron Clusters $SeB_n^{0/-}$ (n = 3–16)

Yue-Ju Yang [1], Shi-Xiong Li [1,*], De-Liang Chen [1] and Zheng-Wen Long [2]

[1] School of Physics and Electronic Science, Guizhou Education University, Guiyang 550018, China
[2] College of physics, Guizhou University, Guiyang 550025, China
* Correspondence: leesxoptics@163.com

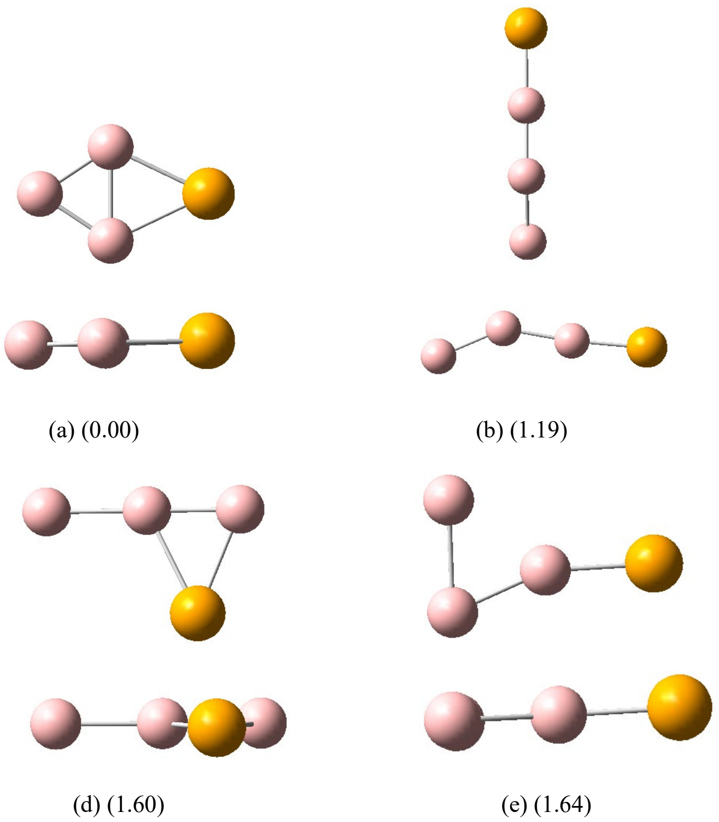

Figure S1. Low-lying isomers of doped boron clusters SeB_3. Values in parentheses are the relative energies (eV) of the five low-energy isomers for SeB_3 at the PBE0/6-311+G* level. The upper row is top view and the bottom row is side view.

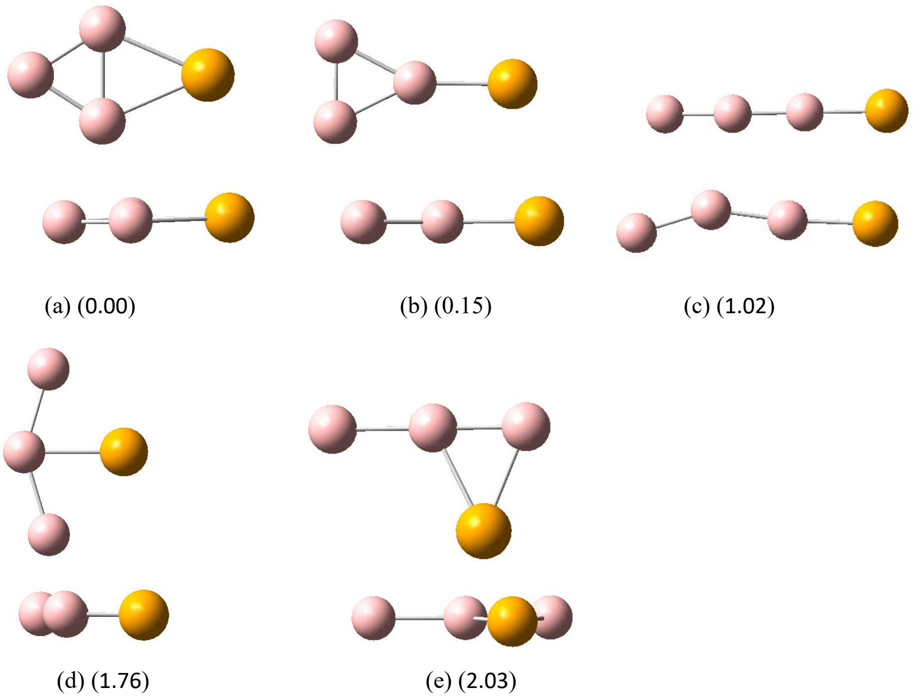

Figure S2. Low-lying isomers of doped boron clusters SeB_3^-. Values in parentheses are the relative energies (eV) of the five low-energy isomers for SeB_3^- at the PBE0/6-311+G* level. The upper row is top view and the bottom row is side view.

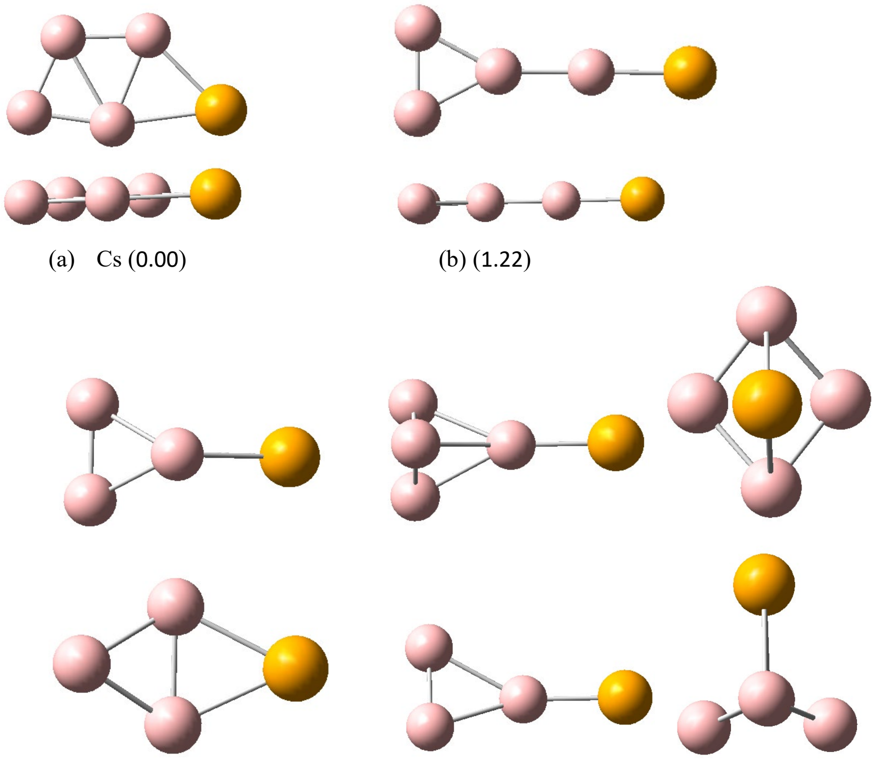

(a) Cs (0.00) (b) (1.22)

(c) (1.87) (d) (1.89) (e) (2.22)

Figure S3. Low-lying isomers of doped boron clusters SeB_4. Values in parentheses are the relative energies (eV) of the five low-energy isomers for SeB_4 at the PBE0/6-311+G* level. The upper row is top view and the bottom row is side view.

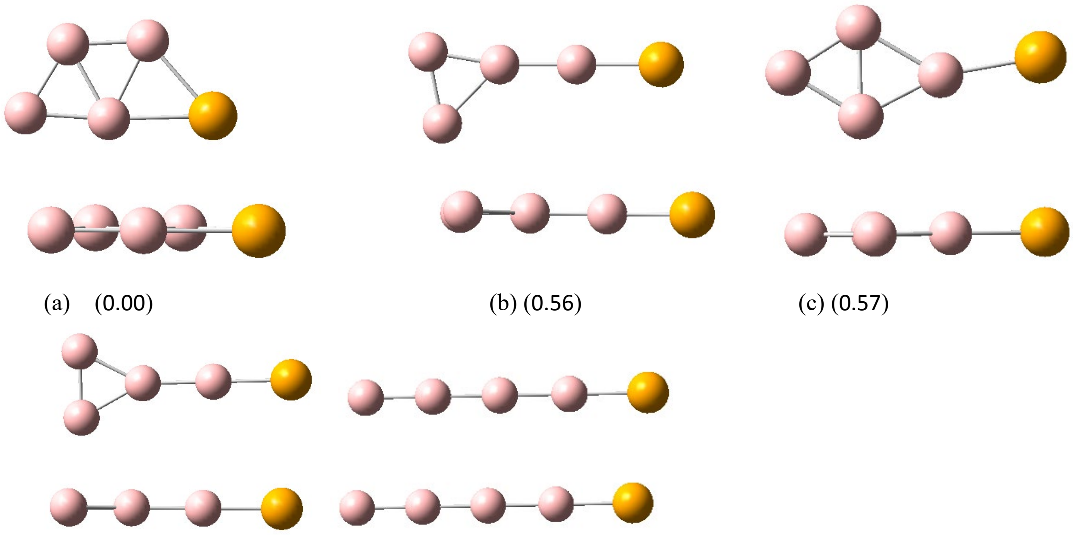

(a) (0.00) (b) (0.56) (c) (0.57)

(d) (0.60) (e) (1.22)

Figure S4. Low-lying isomers of doped boron clusters SeB_4^-. Values in parentheses are the relative energies (eV) of the five low-energy isomers for SeB_4^- at the PBE0/6-311+G* level. The upper row is top view and the bottom row is side view.

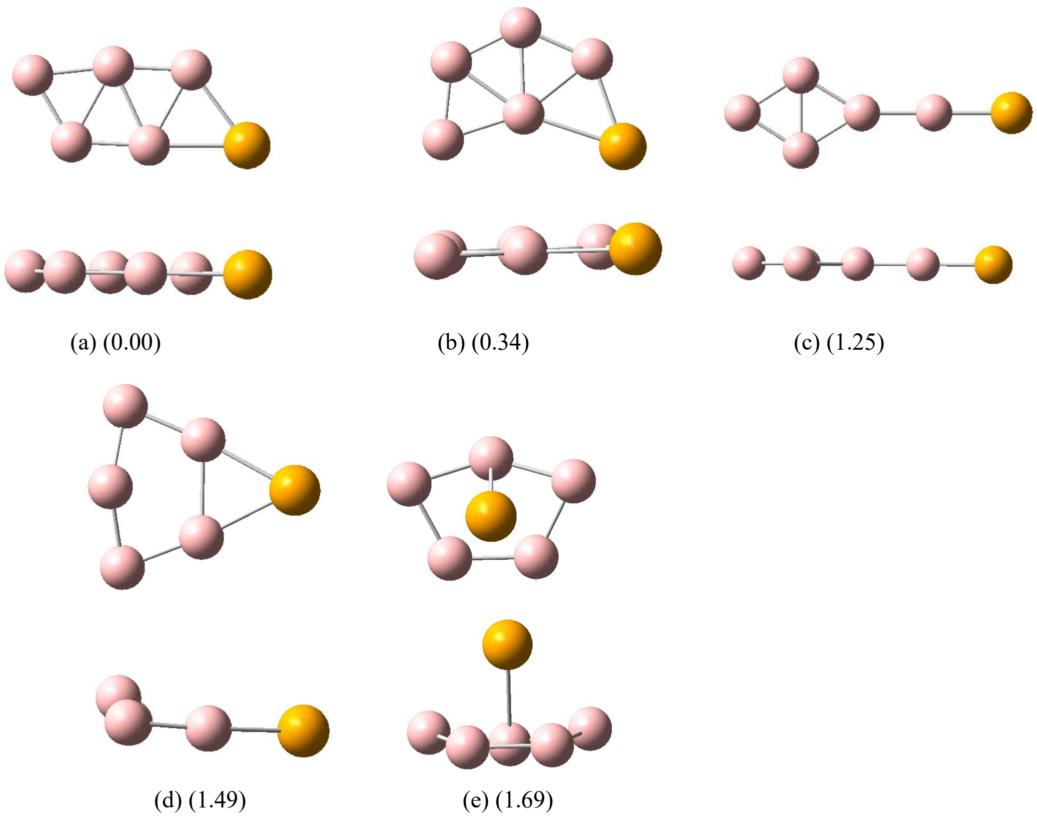

Figure S5. Low-lying isomers of doped boron clusters SeB_5. Values in parentheses are the relative energies (eV) of the five low-energy isomers for SeB_5 at the PBE0/6-311+G* level. The upper row is top view and the bottom row is side view.

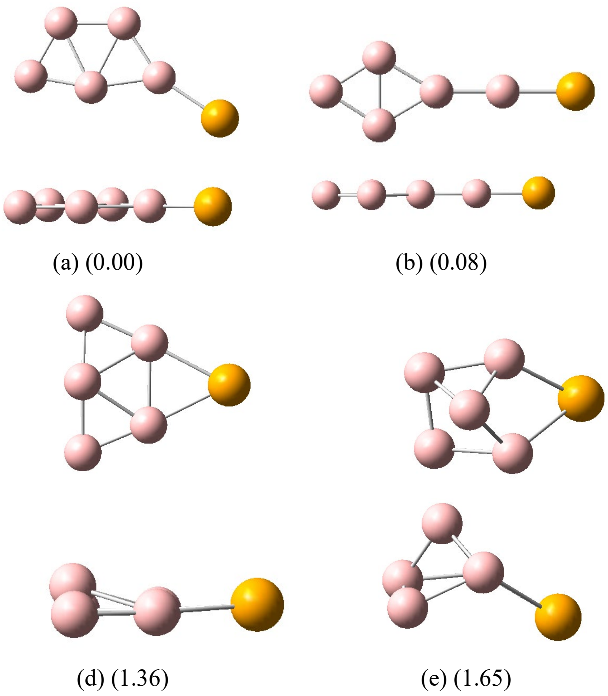

Figure S6. Low-lying isomers of doped boron clusters SeB_5^-. Values in parentheses are the relative energies (eV) of the five low-energy isomers for SeB_5^- at the PBE0/6-311+G* level. The upper row is top view and the bottom row is side view.

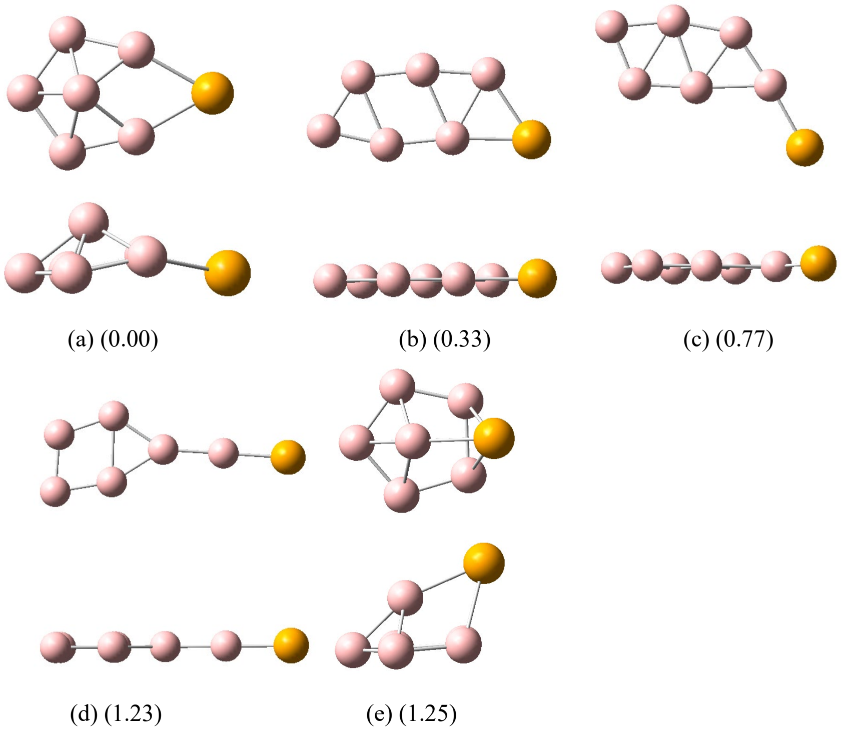

Figure S7. Low-lying isomers of doped boron clusters SeB_6. Values in parentheses are the relative energies (eV) of the five low-energy isomers for SeB_6 at the PBE0/6-311+G* level. The upper row is top view and the bottom row is side view.

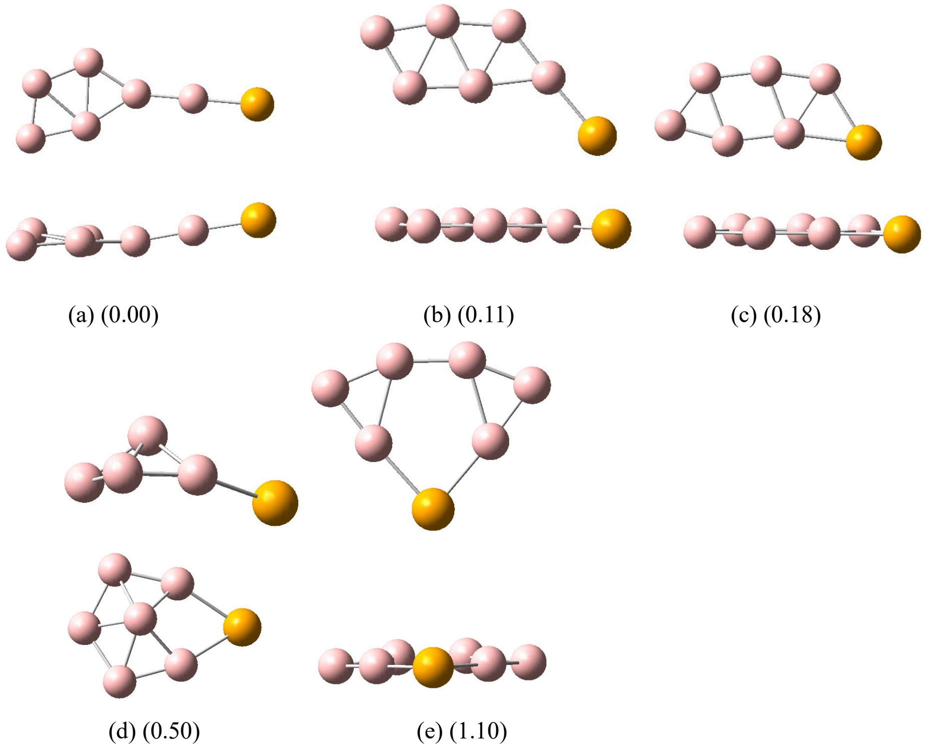

Figure S8. Low-lying isomers of doped boron clusters SeB_6^-. Values in parentheses are the relative energies (eV) of the five low-energy isomers for SeB_6^- at the PBE0/6-311+G* level. The upper row is top view and the bottom row is side view.

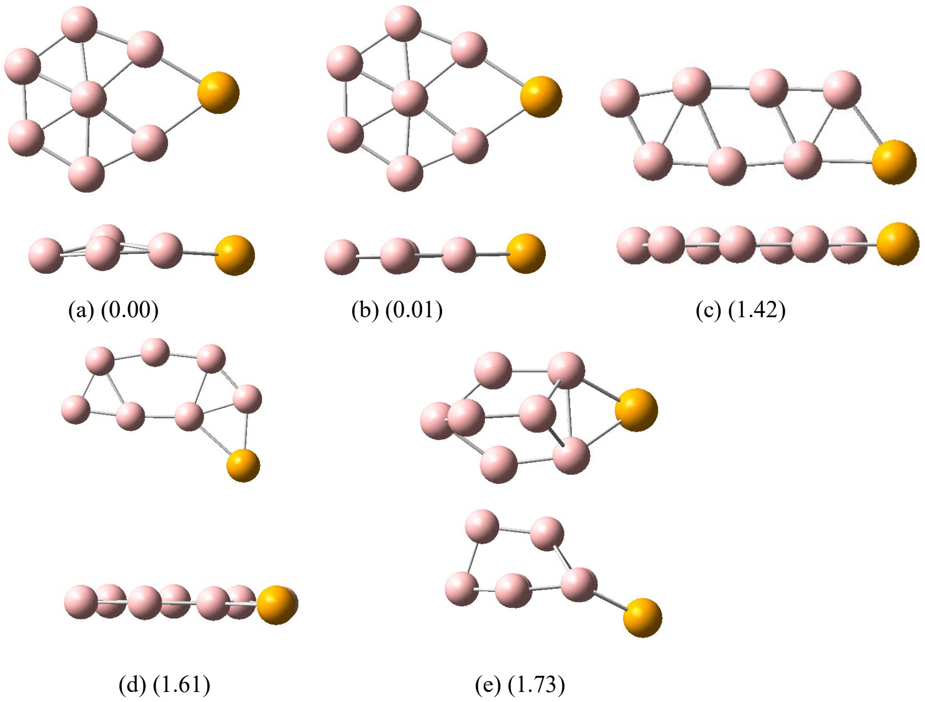

Figure S9. Low-lying isomers of doped boron clusters SeB_7. Values in parentheses are the relative energies (eV) of the five low-energy isomers for SeB_7 at the PBE0/6-311+G* level. The upper row is top view and the bottom row is side view.

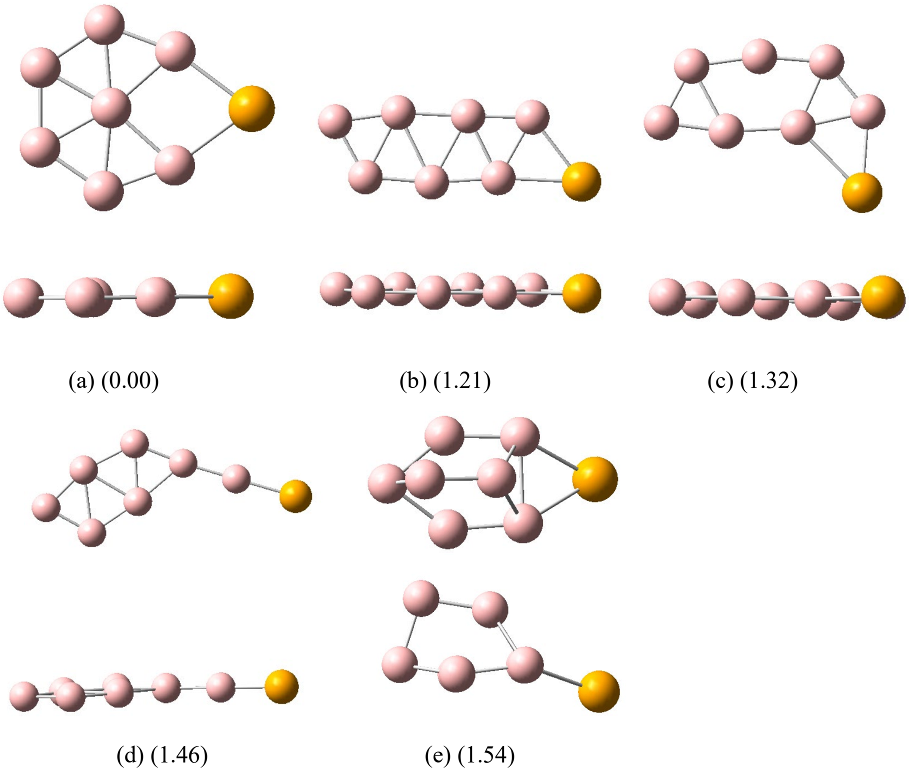

Figure S10. Low-lying isomers of doped boron clusters SeB_7^-. Values in parentheses are the relative energies (eV) of the five low-energy isomers for SeB_7^- at the PBE0/6-311+G* level. The upper row is top view and the bottom row is side view.

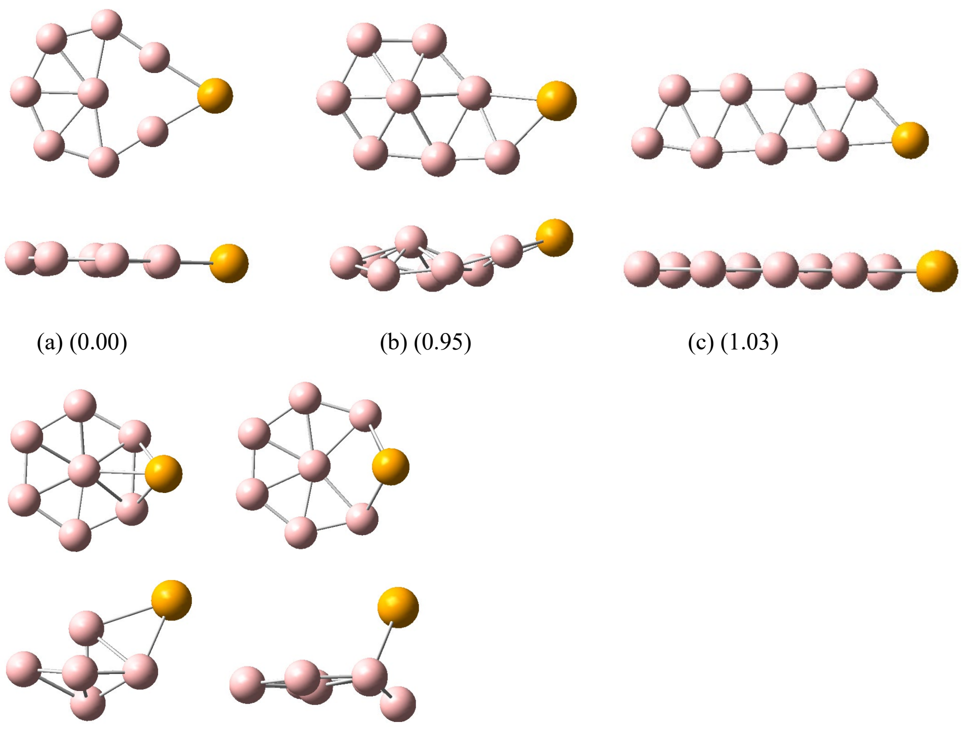

(a) (0.00) (b) (0.95) (c) (1.03)

(d) (1.24) (e) (1.27)

Figure S11. Low-lying isomers of doped boron clusters SeB_8. Values in parentheses are the relative energies (eV) of the five low-energy isomers for SeB_8 at the PBE0/6-311+G* level. The upper row is top view and the bottom row is side view.

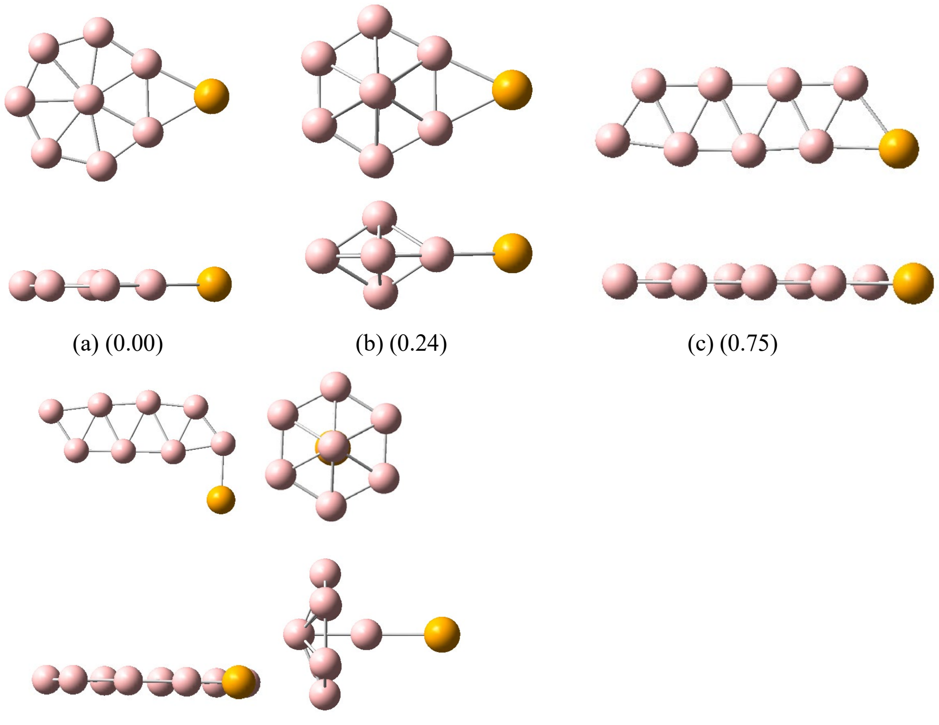

(a) (0.00) (b) (0.24) (c) (0.75)

(d) (1.05) (e) (1.12)

Figure S12. Low-lying isomers of doped boron clusters SeB_8^-. Values in parentheses are the relative energies (eV) of the five low-energy isomers for SeB_8^- at the PBE0/6-311+G* level. The upper row is top view and the bottom row is side view.

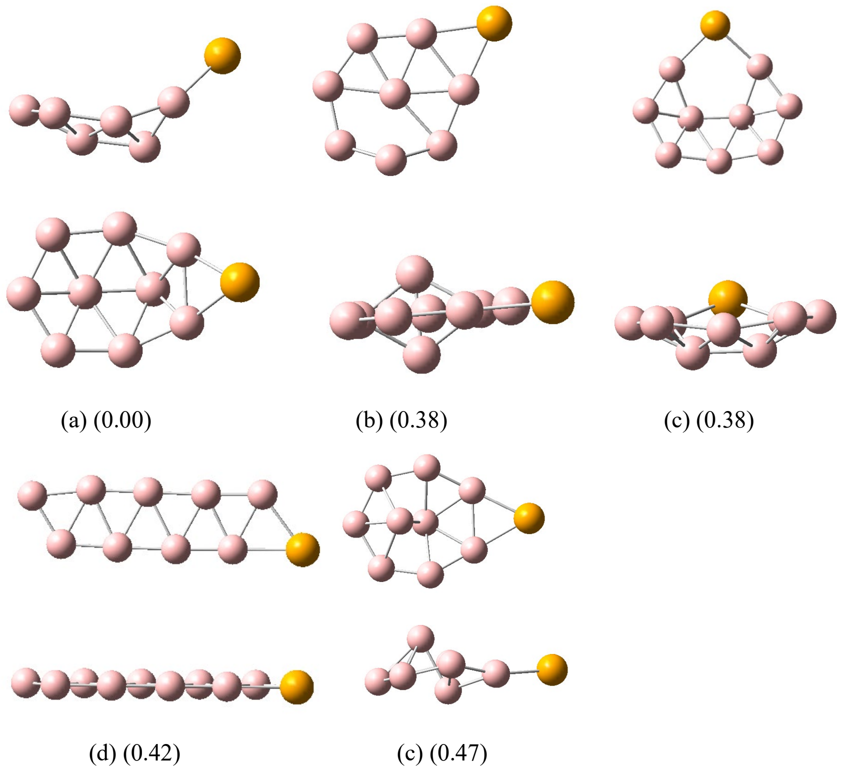

Figure S13. Low-lying isomers of doped boron clusters SeB_9. Values in parentheses are the relative energies (eV) of the five low-energy isomers for SeB_9 at the PBE0/6-311+G* level. The upper row is top view and the bottom row is side view.

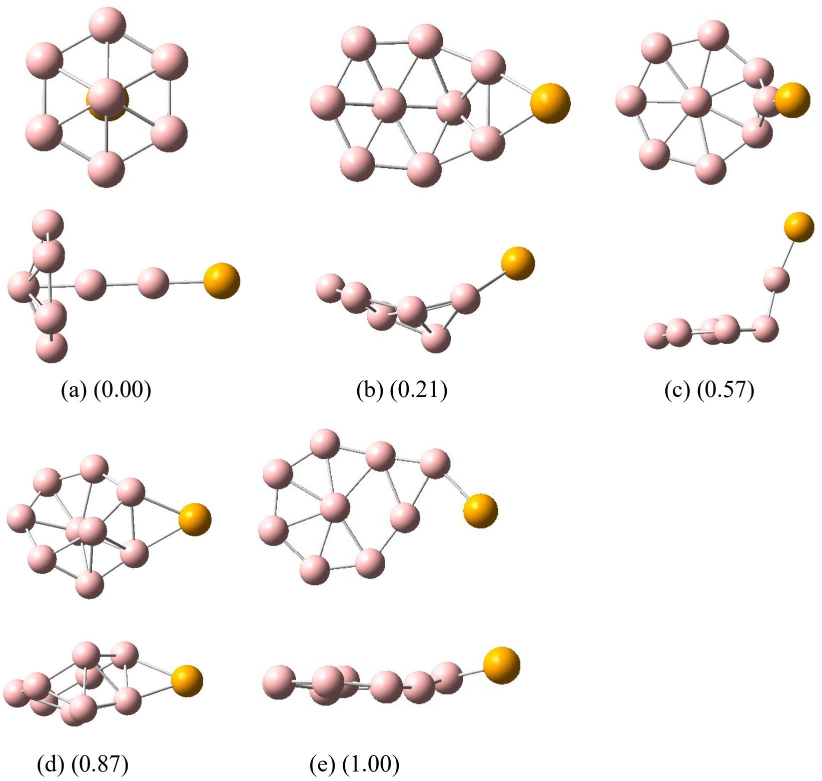

Figure S14. Low-lying isomers of doped boron clusters SeB_9^-. Values in parentheses are the relative energies (eV) of the five low-energy isomers for SeB_9^- at the PBE0/6-311+G* level. The upper row is top view and the bottom row is side view.

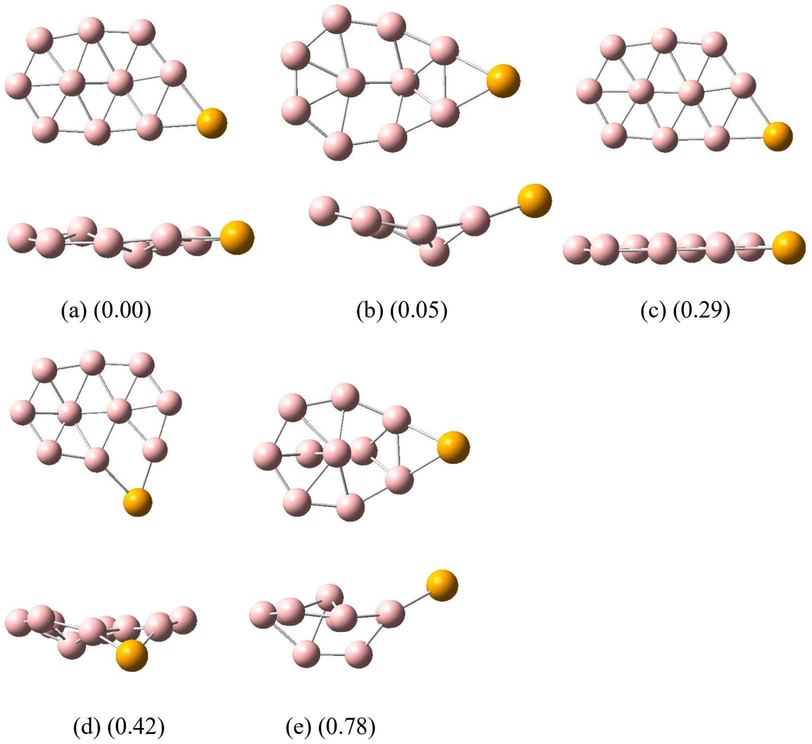

Figure S15. Low-lying isomers of doped boron clusters SeB_{10}. Values in parentheses are the relative energies (eV) of the five low-energy isomers for SeB_{10} at the PBE0/6-311+G* level. The upper row is top view and the bottom row is side view.

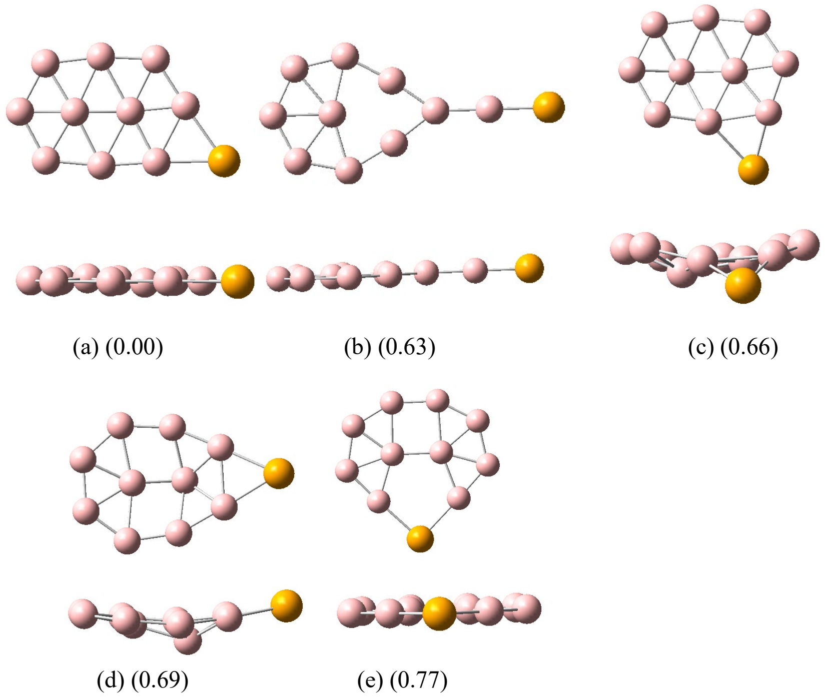

Figure S16. Low-lying isomers of doped boron clusters SeB_{10}^{-}. Values in parentheses are the relative energies (eV) of the five low-energy isomers for SeB_{10}^{-} at the PBE0/6-311+G* level. The upper row is top view and the bottom row is side view.

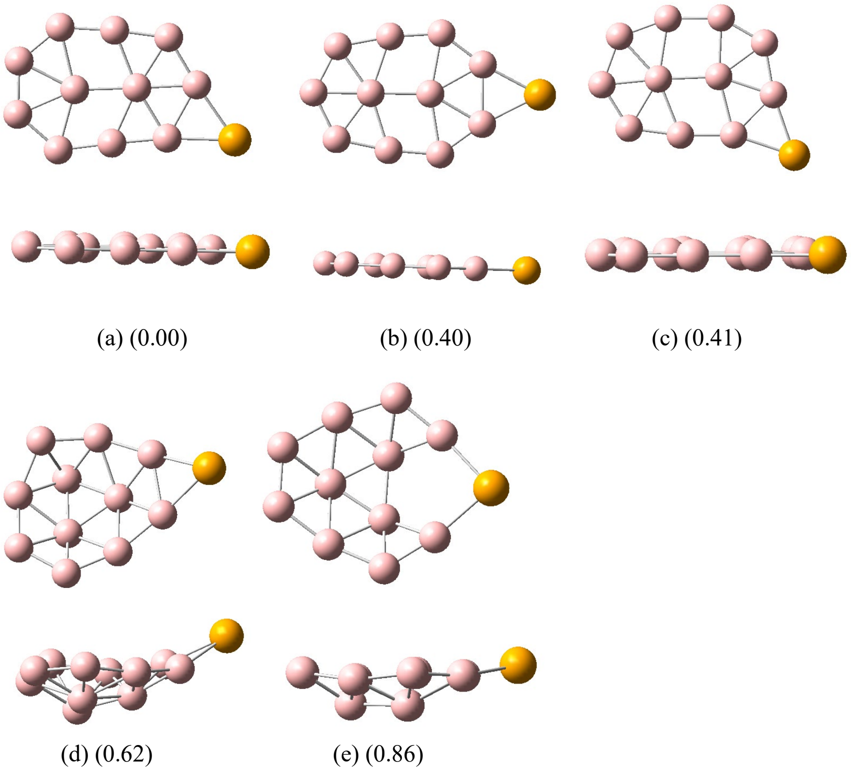

Figure S17. Low-lying isomers of doped boron clusters SeB_{11}. Values in parentheses are the relative energies (eV) of the five low-energy isomers for SeB_{11} at the PBE0/6-311+G* level. The upper row is top view and the bottom row is side view.

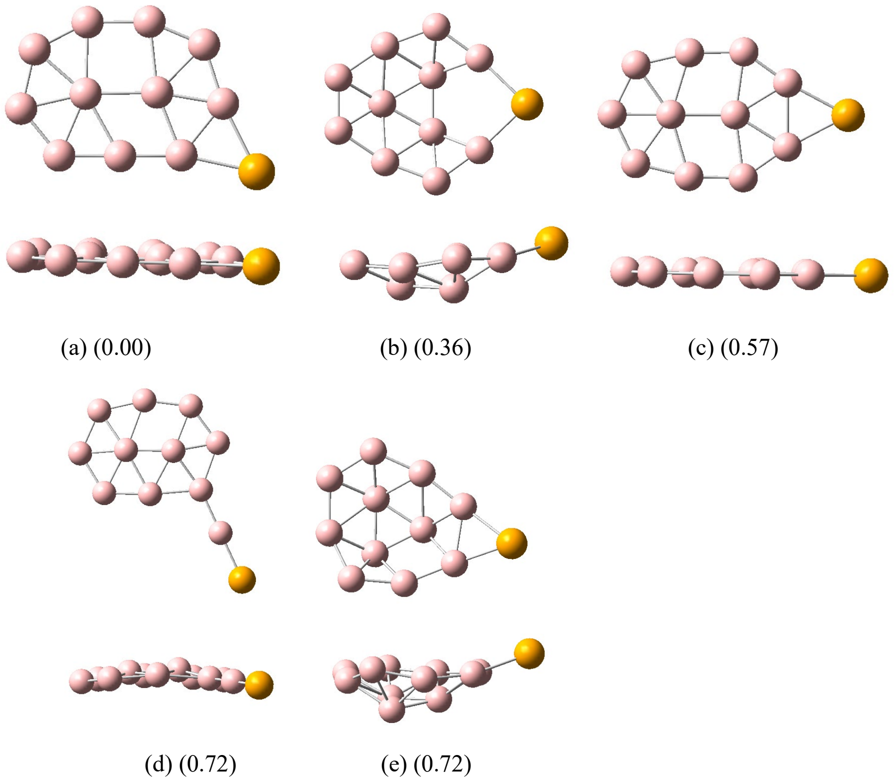

Figure S18. Low-lying isomers of doped boron clusters SeB_{11}^{-}. Values in parentheses are the relative energies (eV) of the five low-energy isomers for SeB_{11}^{-} at the PBE0/6-311+G* level. The upper row is top view and the bottom row is side view.

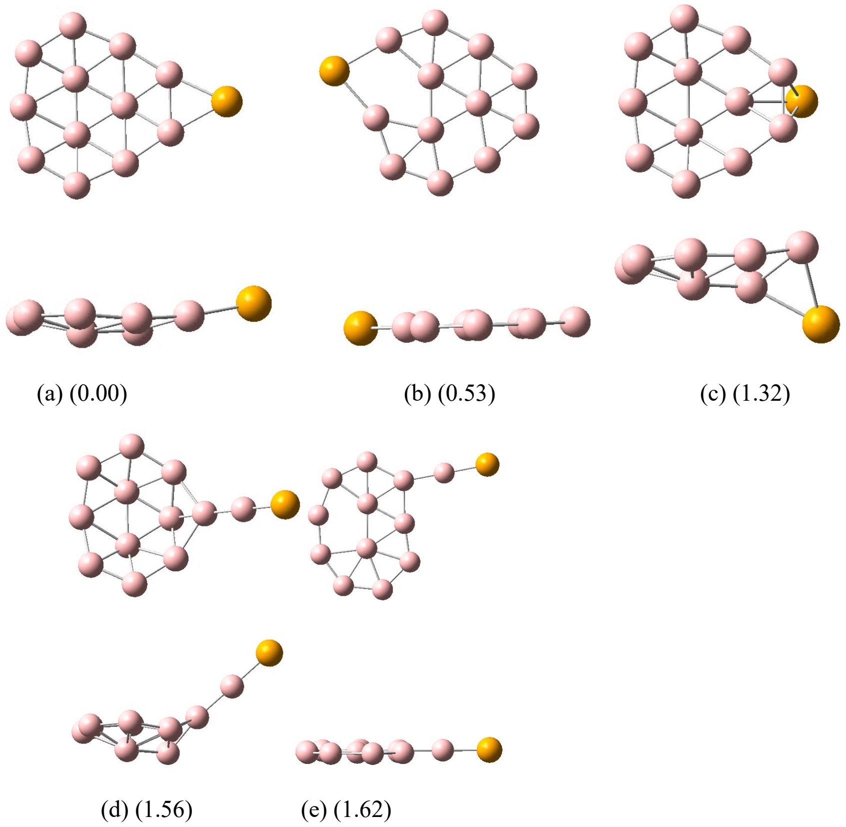

Figure S19. Low-lying isomers of doped boron clusters SeB_{12}. Values in parentheses are the relative energies (eV) of the five low-energy isomers for SeB_{12} at the PBE0/6-311+G* level. The upper row is top view and the bottom row is side view.

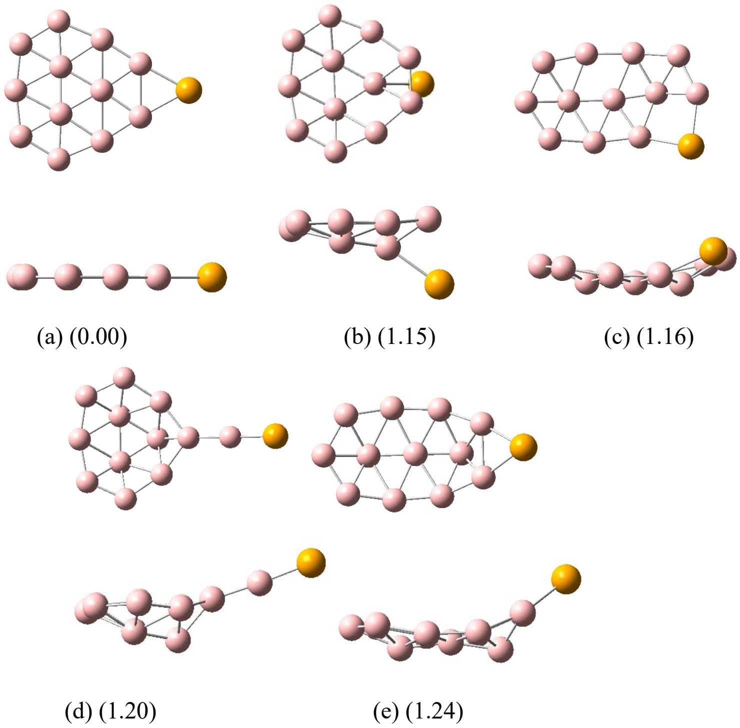

Figure S20. Low-lying isomers of doped boron clusters SeB_{12}^{-}. Values in parentheses are the relative energies (eV) of the five low-energy isomers for SeB_{12}^{-} at the PBE0/6-311+G* level. The upper row is top view and the bottom row is side view.

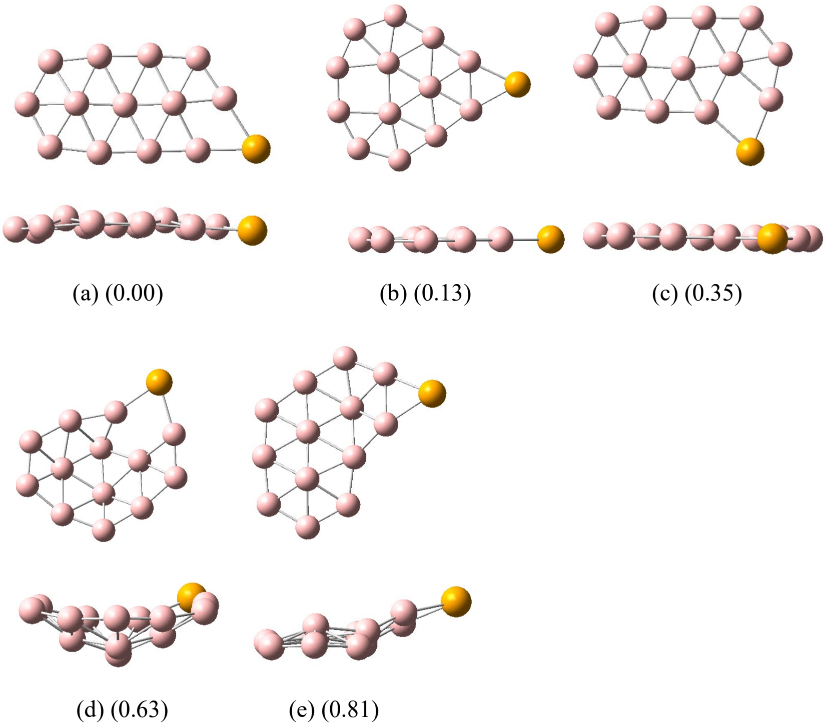

Figure S21. Low-lying isomers of doped boron clusters SeB_{13}. Values in parentheses are the relative energies (eV) of the five low-energy isomers for SeB_{13} at the PBE0/6-311+G* level. The upper row is top view and the bottom row is side view.

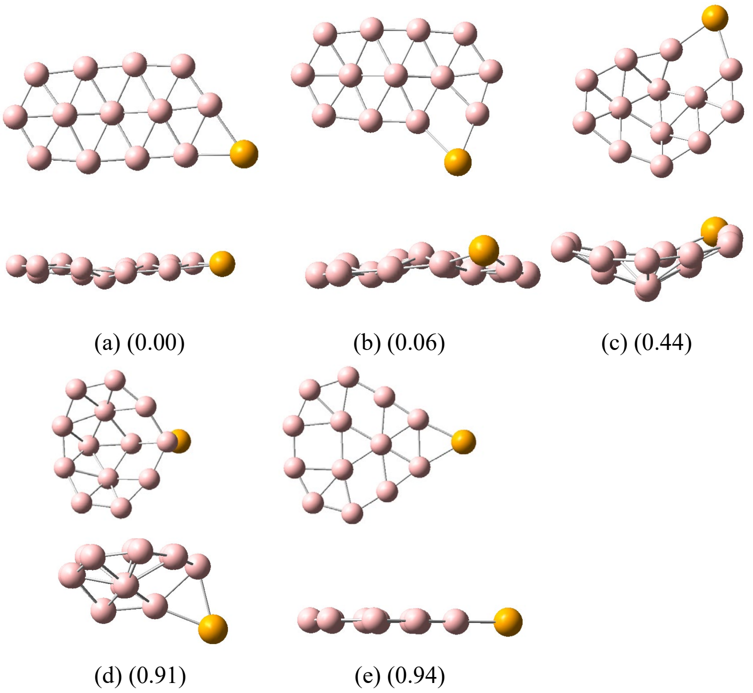

Figure S22. Low-lying isomers of doped boron clusters SeB_{13}^{-}. Values in parentheses are the relative energies (eV) of the five low-energy isomers for SeB_{13}^{-} at the PBE0/6-311+G* level. The upper row is top view and the bottom row is side view.

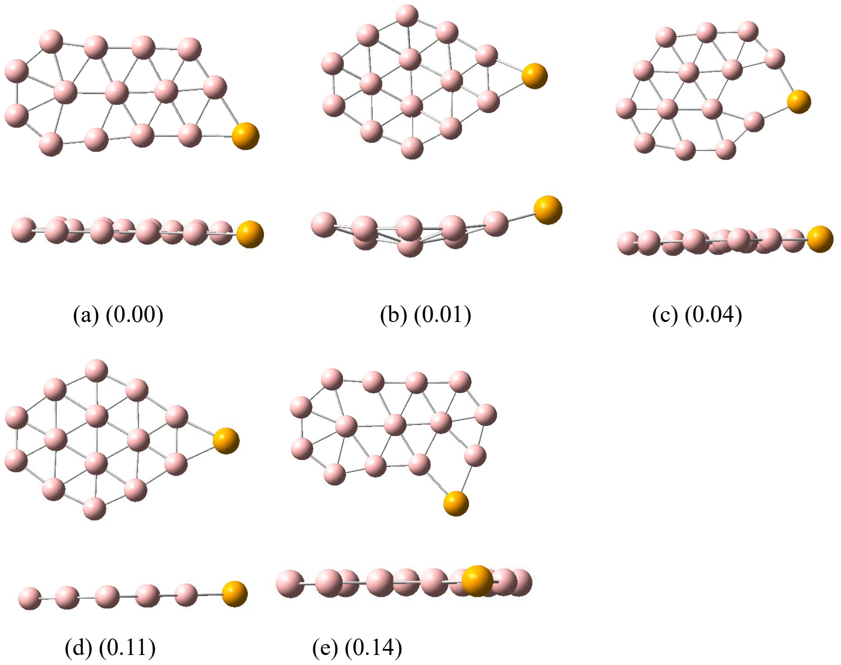

Figure S23. Low-lying isomers of doped boron clusters SeB_{14}. Values in parentheses are the relative energies (eV) of the five low-energy isomers for SeB_{14} at the PBE0/6-311+G* level. The upper row is top view and the bottom row is side view.

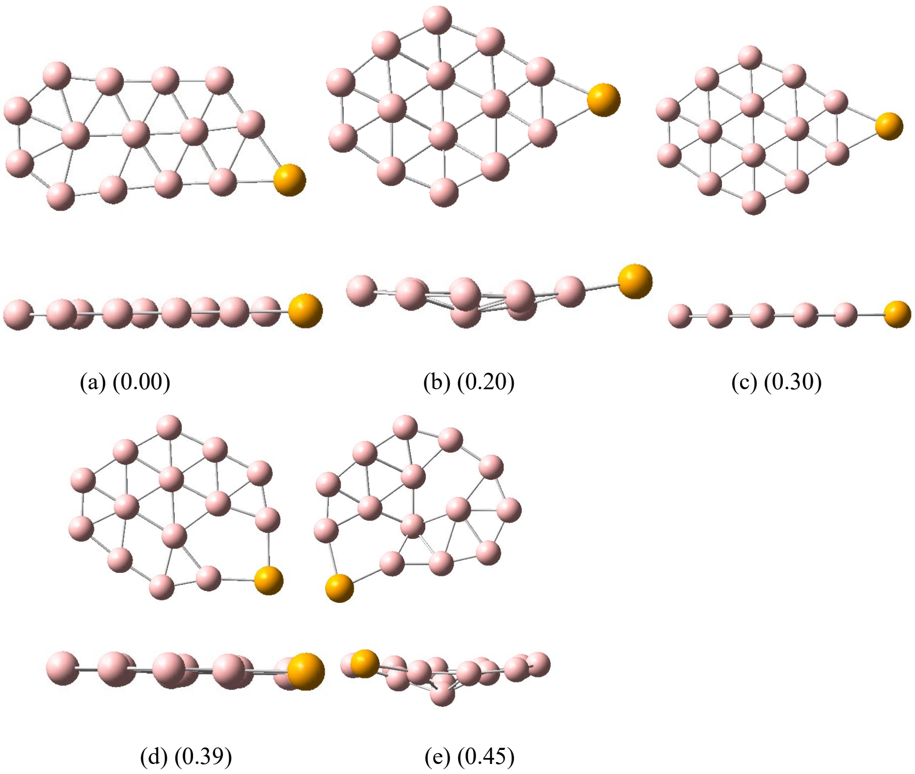

Figure S24. Low-lying isomers of doped boron clusters SeB_{14}^{-}. Values in parentheses are the relative energies (eV) of the five low-energy isomers for SeB_{14}^{-} at the PBE0/6-311+G* level. The upper row is top view and the bottom row is side view.

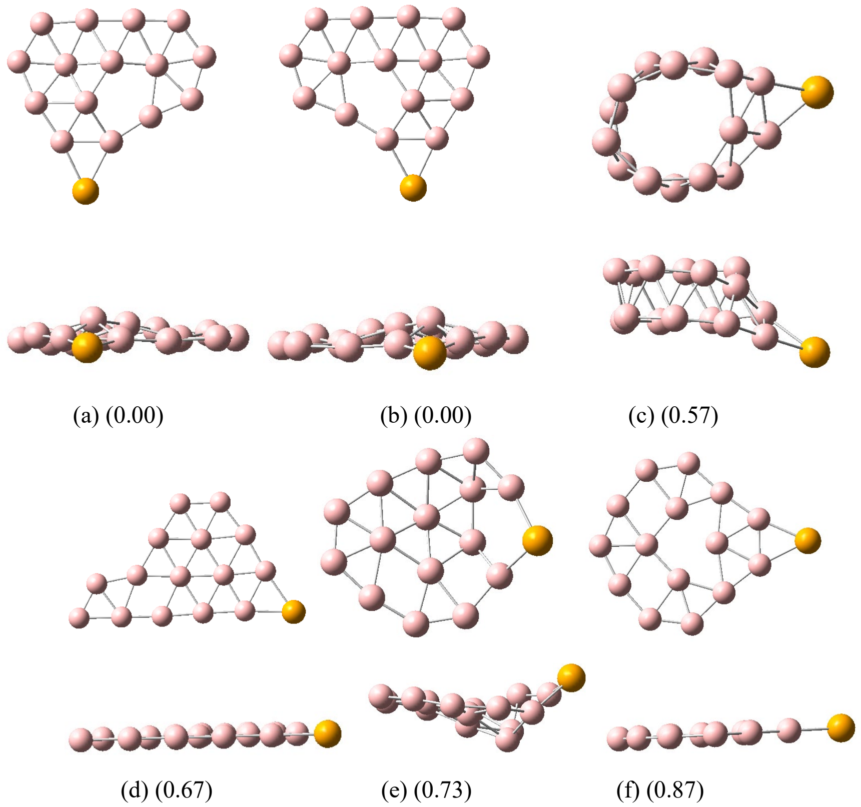

Figure S25. Low-lying isomers of doped boron clusters SeB_{15}. Values in parentheses are the relative energies (eV) of the five low-energy isomers for SeB_{15} at the PBE0/6-311+G* level. The upper row is top view and the bottom row is side view.

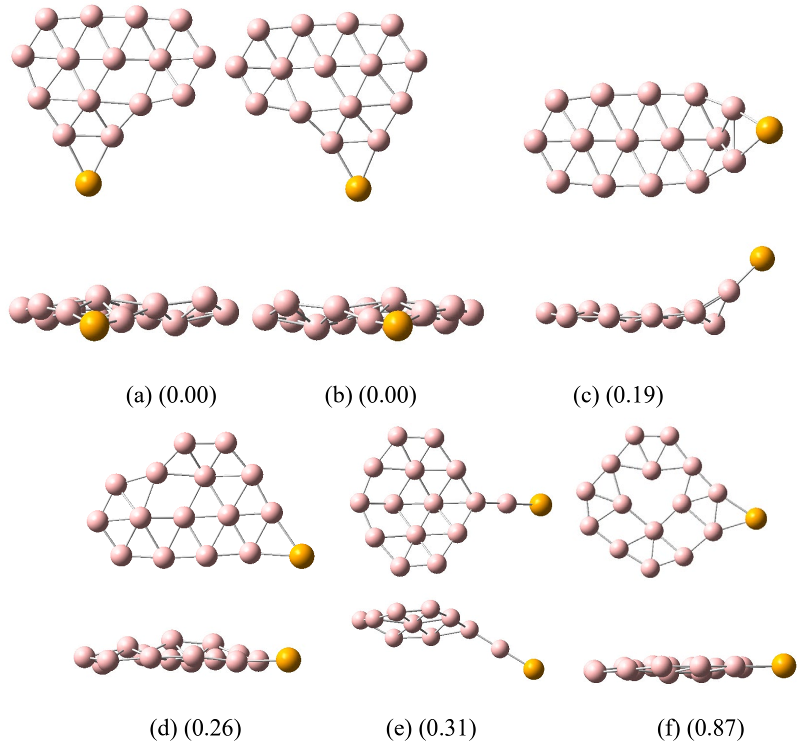

Figure S26. Low-lying isomers of doped boron clusters SeB_{15}^{-}. Values in parentheses are the relative energies (eV) of the five low-energy isomers for SeB_{15}^{-} at the PBE0/6-311+G* level. The upper row is top view and the bottom row is side view.

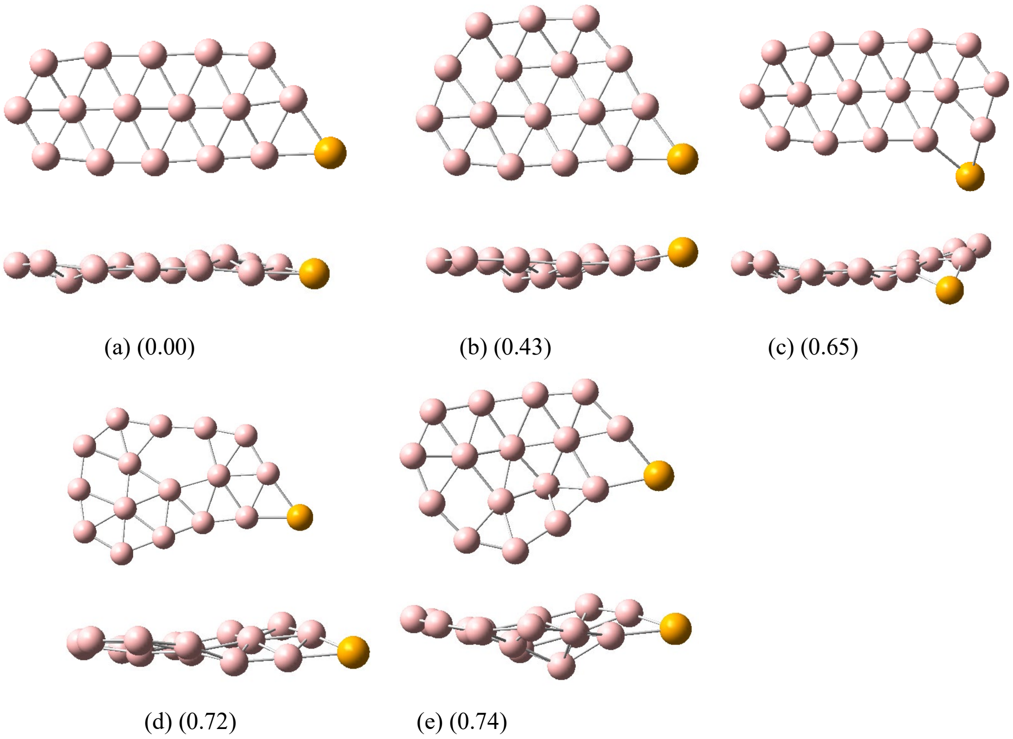

Figure S27. Low-lying isomers of doped boron clusters SeB_{16}. Values in parentheses are the relative energies (eV) of the five low-energy isomers for SeB_{16} at the PBE0/6-311+G* level. The upper row is top view and the bottom row is side view.

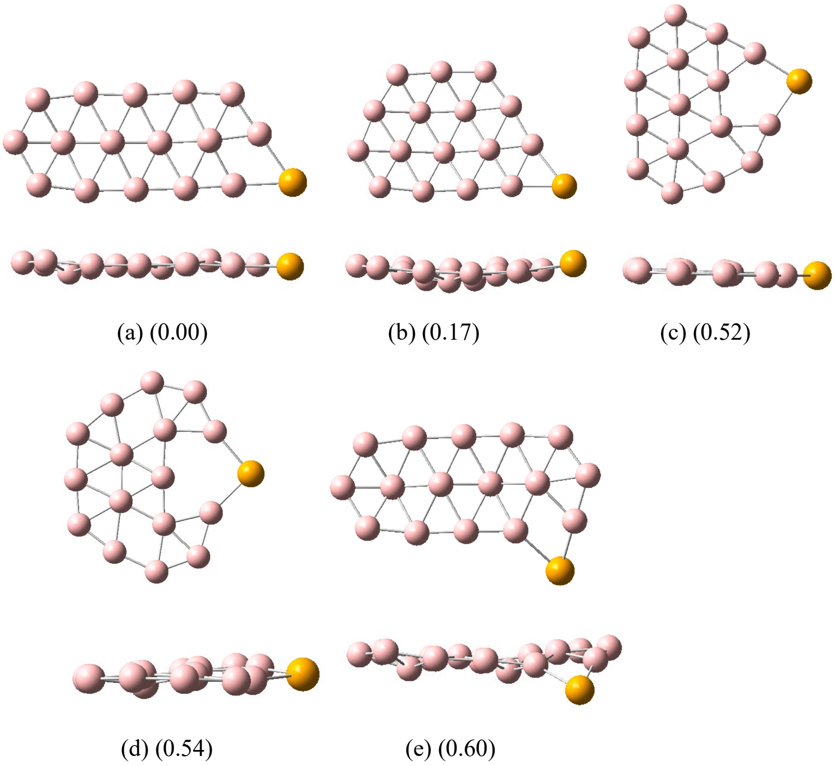

Figure S28. Low-lying isomers of doped boron clusters SeB_{16}^{-}. Values in parentheses are the relative energies (eV) of the five low-energy isomers for SeB_{16}^{-} at the PBE0/6-311+G* level. The upper row is top view and the bottom row is side view.

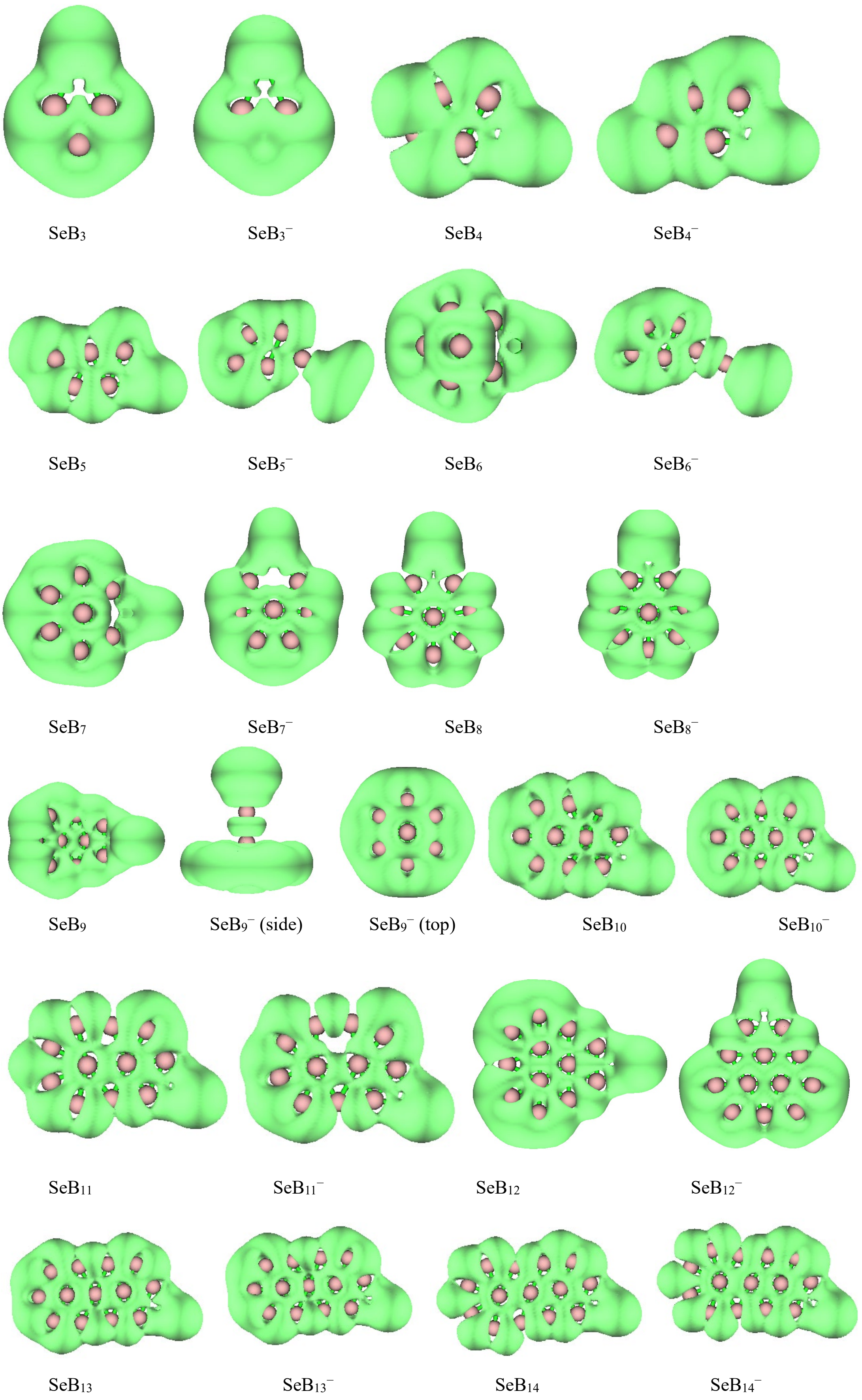
SeB3
SeB3−
SeB4
SeB4−
SeB5
SeB5−
SeB6
SeB6−
SeB7
SeB7−
SeB8
SeB8−
SeB9
SeB9− (side)
SeB9− (top)
SeB10
SeB10−
SeB11
SeB11−
SeB12
SeB12−
SeB13
SeB13−
SeB14
SeB14−

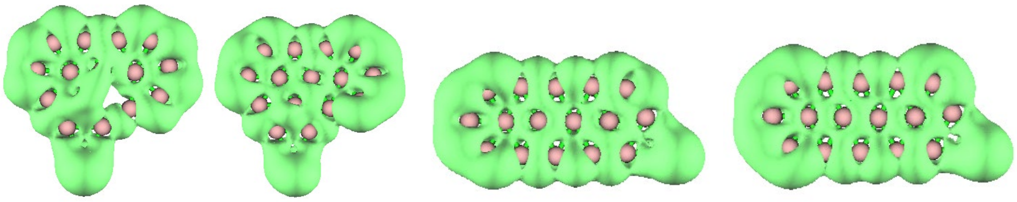

SeB_{15} SeB_{15}^{-} SeB_{16} SeB_{16}^{-}

Figure S29. Electron localization function (ELF) with the isovalue set to 0.60.

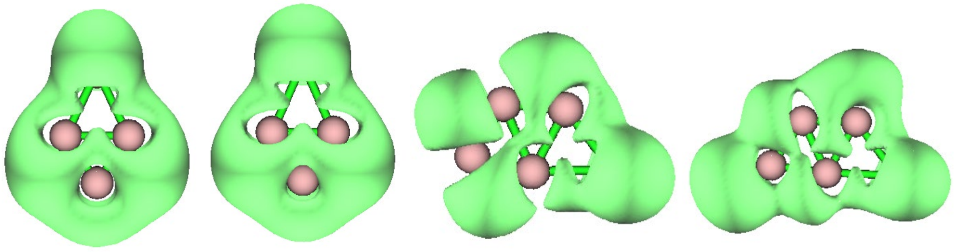

SeB_3 SeB_3^{-} SeB_4 SeB_4^{-}

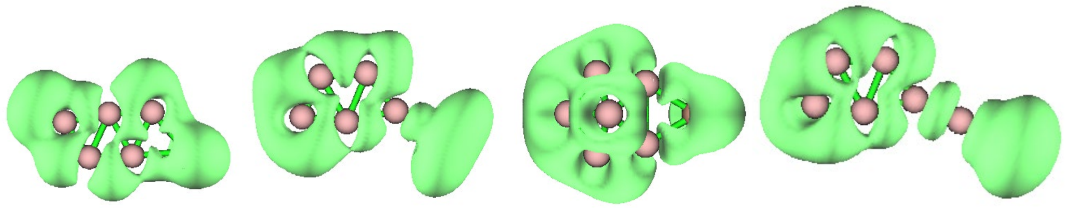

SeB_5 SeB_5^{-} SeB_6 SeB_6^{-}

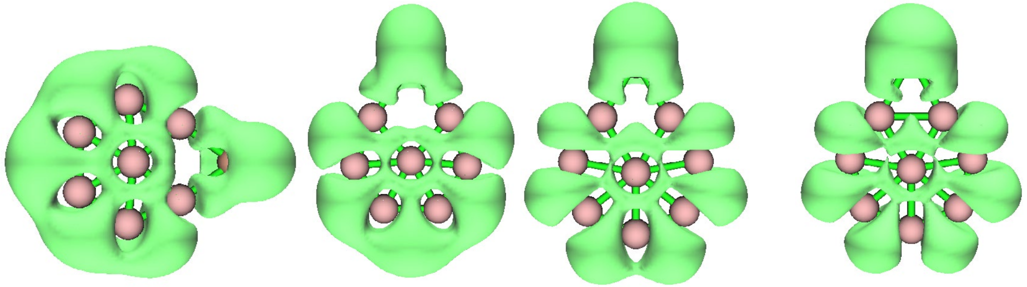

SeB_7 SeB_7^{-} SeB_8 SeB_8^{-}

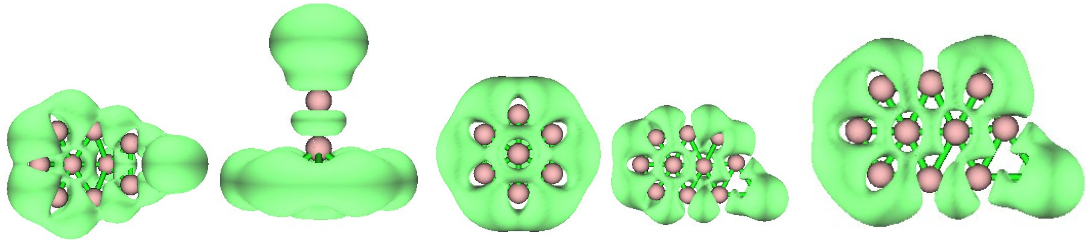

SeB_9 SeB_9^{-} (side) SeB_9^{-} (top) SeB_{10} SeB_{10}^{-}

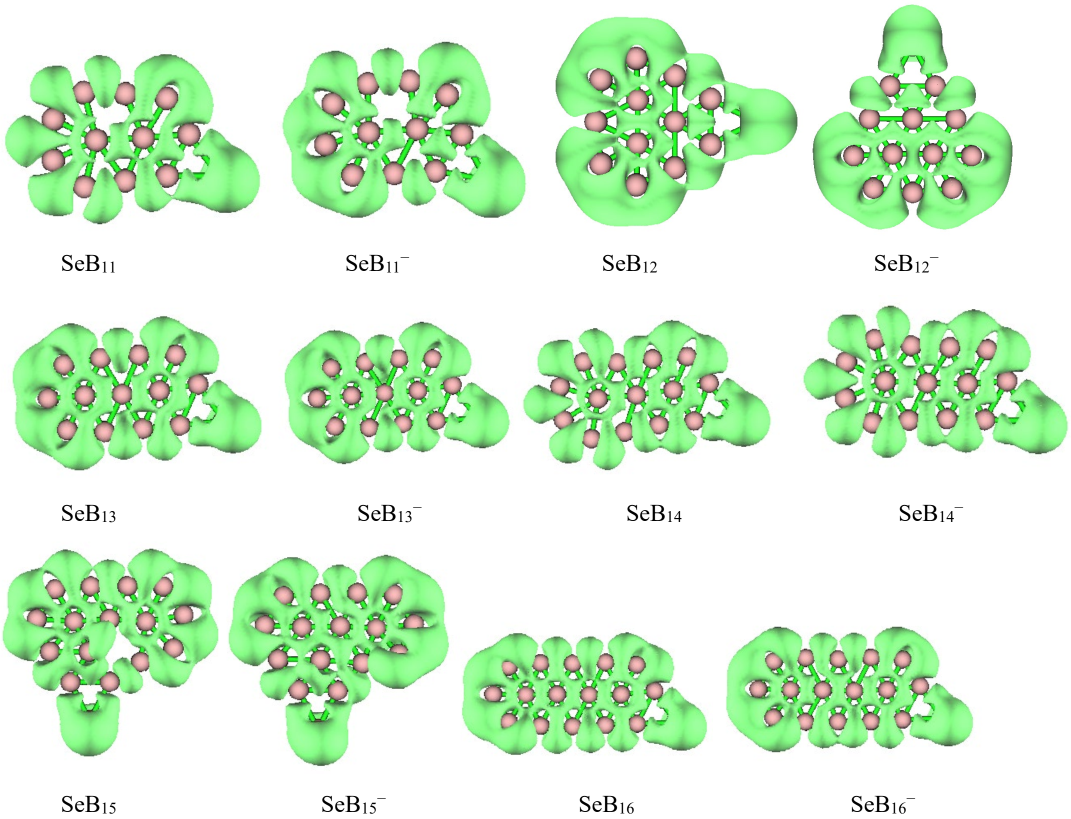

Figure S30. Electron localization function (ELF) with the isovalue set to 0.70.

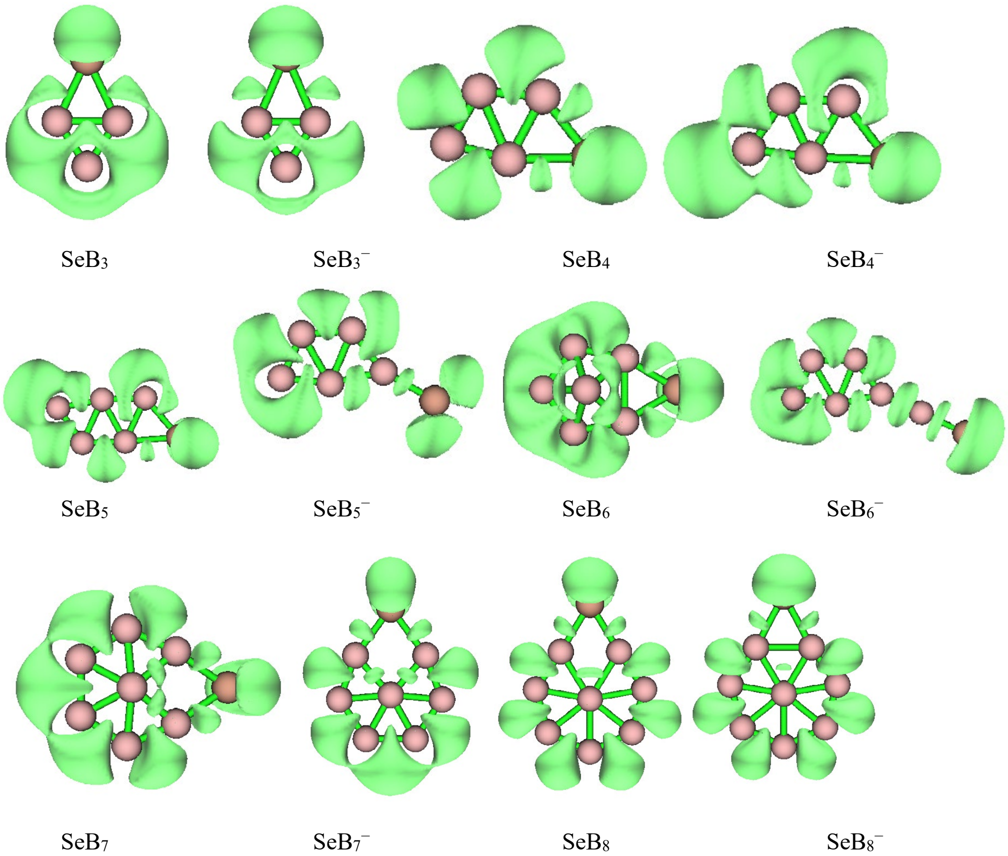

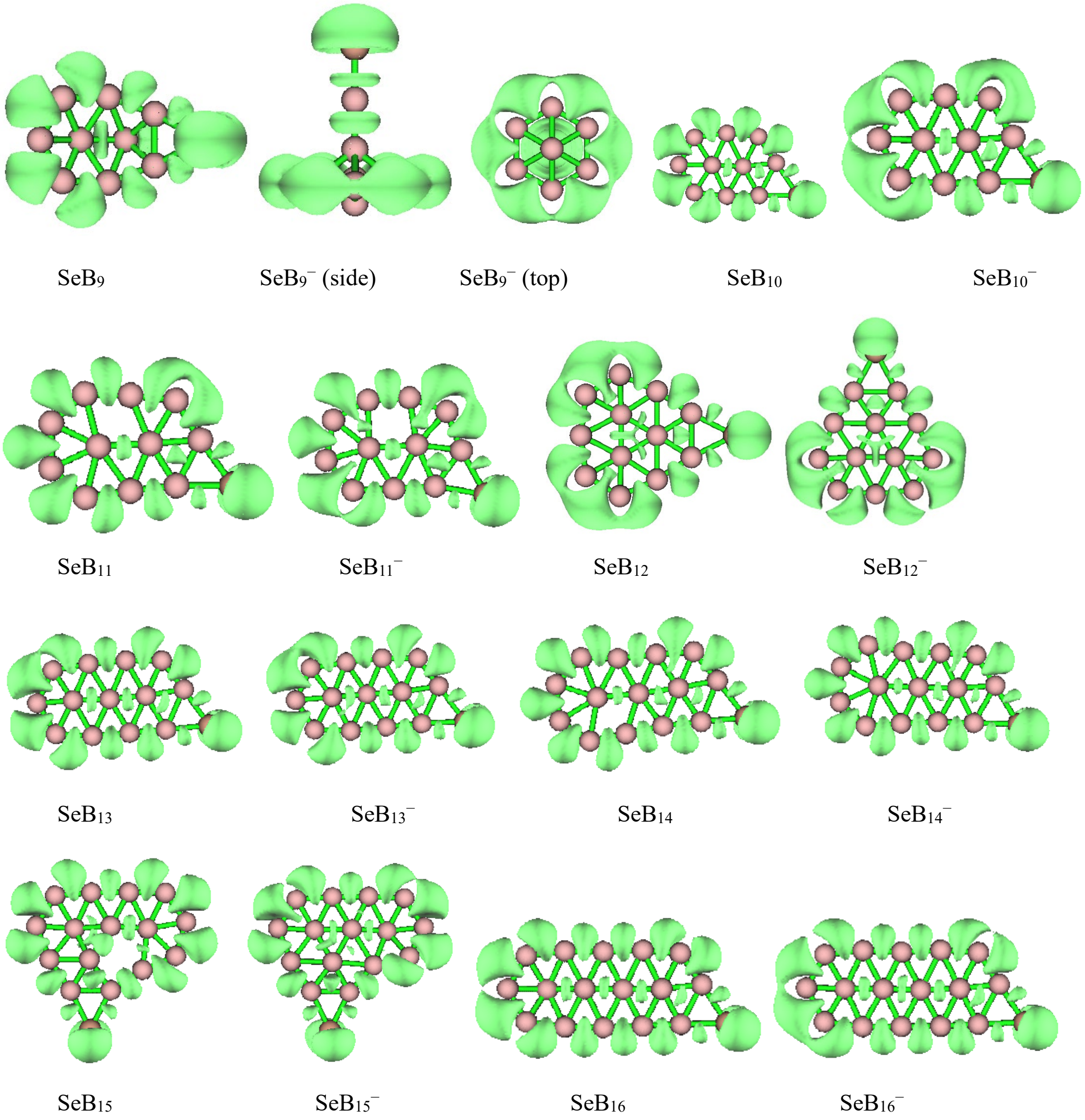

Figure S31. Electron localization function (ELF) with the isovalue set to 0.80.

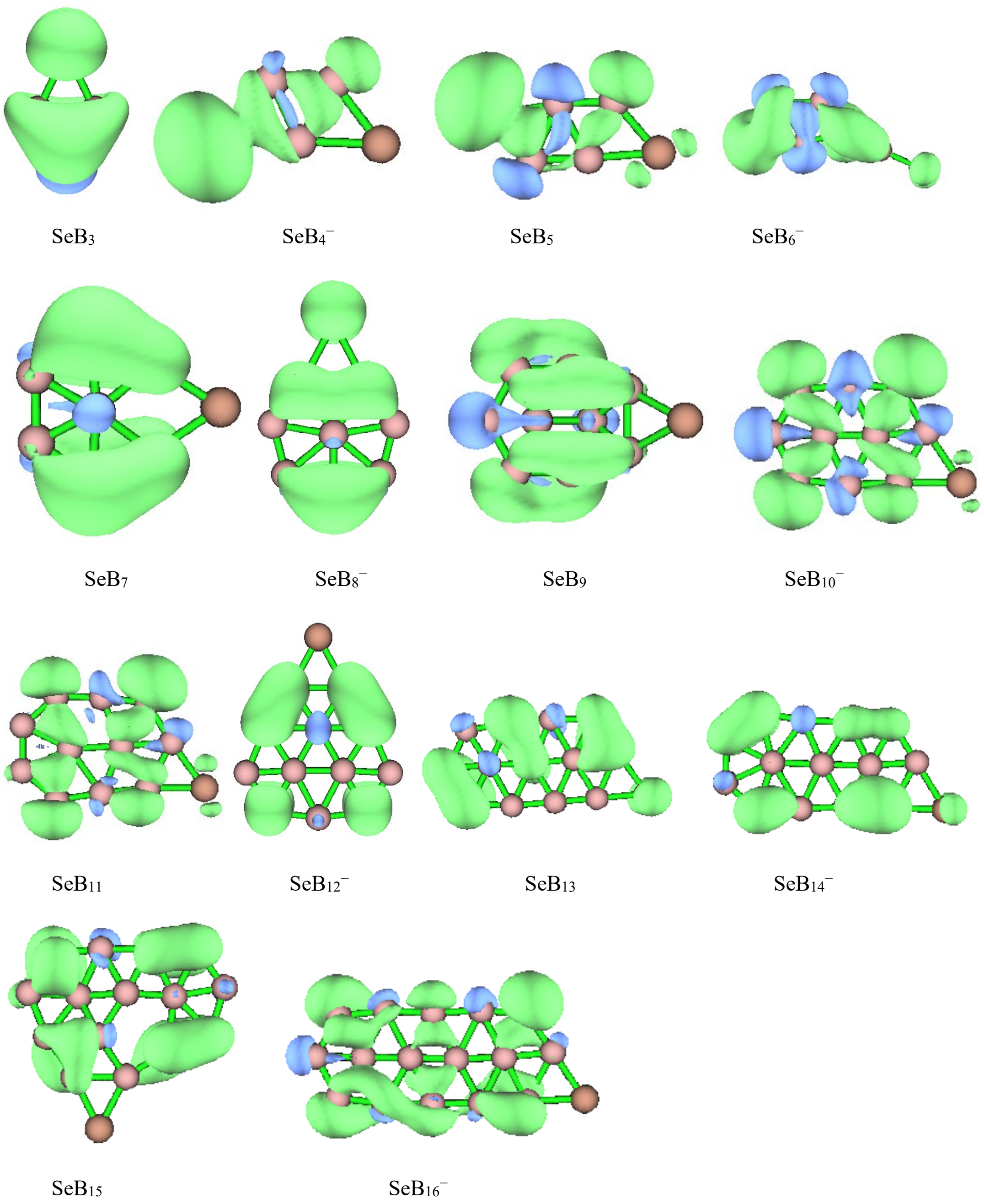

Figure S32. Spin density with the isovalue set to 0.002. Green represents a positive value (alpha electrons), and blue represents a negative value (beta electrons).
